# Stepwise use of genomics and transcriptomics technologies increases diagnostic yield in Mendelian disorders

**DOI:** 10.3389/fcell.2023.1021920

**Published:** 2023-02-28

**Authors:** Estelle Colin, Yannis Duffourd, Martin Chevarin, Emilie Tisserant, Simon Verdez, Julien Paccaud, Ange-Line Bruel, Frédéric Tran Mau-Them, Anne-Sophie Denommé-Pichon, Julien Thevenon, Hana Safraou, Thomas Besnard, Alice Goldenberg, Benjamin Cogné, Bertrand Isidor, Julian Delanne, Arthur Sorlin, Sébastien Moutton, Mélanie Fradin, Christèle Dubourg, Magali Gorce, Dominique Bonneau, Salima El Chehadeh, François-Guillaume Debray, Martine Doco-Fenzy, Kevin Uguen, Nicolas Chatron, Bernard Aral, Nathalie Marle, Paul Kuentz, Anne Boland, Robert Olaso, Jean-François Deleuze, Damien Sanlaville, Patrick Callier, Christophe Philippe, Christel Thauvin-Robinet, Laurence Faivre, Antonio Vitobello

**Affiliations:** ^1^ UFR Des Sciences de Santé, INSERM-Université de Bourgogne UMR1231 GAD “Génétique des Anomalies du Développement”, FHUTRANSLAD, Dijon, France; ^2^ Service de Génétique Médicale, CHU d’Angers, Angers, France; ^3^ Unité Fonctionnelle Innovation en Diagnostic Génomique des Maladies Rares, FHU-TRANSLAD, CHU Dijon Bourgogne, Dijon, France; ^4^ Service de Génétique Médicale, Nantes Université, CHU Nantes, Nantes, France; ^5^ CNRS, INSERM, L’institut du thorax, Nantes Université, CHU Nantes, Nantes, France; ^6^ Department of Genetics and Reference Center for Developmental Disorders, Normandy Center for Genomic and Personalized Medicine, Rouen University Hospital, Rouen, France; ^7^ Normandie Univ, UNIROUEN, Inserm U1245, Rouen, France; ^8^ Service de Génétique Médicale, CHU de Nantes, Nantes, France; ^9^ Centre de Génétique et Centre de référence “Anomalies du Développement et Syndromes Malformatifs”, Hôpital d’Enfants, Centre Hospitalier Universitaire de Dijon, Dijon, France; ^10^ CHU Rennes, Service de Génétique Clinique, Centre de Référence Maladies Rares, CLAD-Ouest, Rennes, France; ^11^ Service de Génétique Moléculaire et Génomique, CHU Rennes, Rennes, France; ^12^ Univ Rennes, CNRS, Institut de Genetique et Developpement de Rennes, UMR 6290, Rennes, France; ^13^ Service de Génétique Médicale, Hôpital de Hautepierre, CHU Strasbourg, Strasbourg, France; ^14^ Metabolic Unit, Department of Human Genetics, CHU of Liege, University of Liege, Liege, Belgium; ^15^ Medical School IFR53, EA3801, Université de Reims Champagne-Ardenne, Reims, France; ^16^ Service de Génétique, CHU Reims, Reims, France; ^17^ Department of Genetics and Reference Center for Developmental Disorders, Lyon University Hospital, Groupement Hospitalier Est, Hospices Civils de Lyon, Lyon, France; ^18^ CHU Brest, Inserm, Univ Brest, EFS, UMR 1078, GGB, Brest, France; ^19^ Laboratoire de Génétique Chromosomique et Moléculaire, Pôle Biologie, CHU de Dijon, Dijon, France; ^20^ Oncobiologie Génétique Bioinformatique, PCBio, Centre Hospitalier Universitaire de Besançon, Besançon, France; ^21^ Université Paris-Saclay, CEA, Centre National de Recherche en Génomique Humaine (CNRGH), Evry, France; ^22^ LabEx GENMED (Medical Genomics), Dijon, France; ^23^ Centre de Référence Maladies Rares “Déficiences Intellectuelles de Causes Rares”, Centre de Génétique, FHU-TRANSLAD, CHU Dijon Bourgogne, Dijon, France

**Keywords:** genome sequencing, RNA-seq, optical genome mapping, long-read sequencing, clinical diagnoses

## Abstract

**Purpose:** Multi-omics offer worthwhile and increasingly accessible technologies to diagnostic laboratories seeking potential second-tier strategies to help patients with unresolved rare diseases, especially patients clinically diagnosed with a rare OMIM (Online Mendelian Inheritance in Man) disease. However, no consensus exists regarding the optimal diagnostic care pathway to adopt after negative results with standard approaches.

**Methods:** In 15 unsolved individuals clinically diagnosed with recognizable OMIM diseases but with negative or inconclusive first-line genetic results, we explored the utility of a multi-step approach using several novel omics technologies to establish a molecular diagnosis. Inclusion criteria included a clinical autosomal recessive disease diagnosis and single heterozygous pathogenic variant in the gene of interest identified by first-line analysis (60%–9/15) or a clinical diagnosis of an X-linked recessive or autosomal dominant disease with no causative variant identified (40%–6/15). We performed a multi-step analysis involving short-read genome sequencing (srGS) and complementary approaches such as mRNA sequencing (mRNA-seq), long-read genome sequencing (lrG), or optical genome mapping (oGM) selected according to the outcome of the GS analysis.

**Results:** SrGS alone or in combination with additional genomic and/or transcriptomic technologies allowed us to resolve 87% of individuals by identifying single nucleotide variants/indels missed by first-line targeted tests, identifying variants affecting transcription, or structural variants sometimes requiring lrGS or oGM for their characterization.

**Conclusion:** Hypothesis-driven implementation of combined omics technologies is particularly effective in identifying molecular etiologies. In this study, we detail our experience of the implementation of genomics and transcriptomics technologies in a pilot cohort of previously investigated patients with a typical clinical diagnosis without molecular etiology.

## 1 Introduction

Rare genetic diseases, which individually affect a relatively small number of people, collectively represent a public health concern, with more than 300 million people affected worldwide (Boycott et al., 2019). The molecular diagnosis of such conditions has a profound public impact involving genetic counseling and treatment to improve disease management (Sawyer et al., 2016; Hartley et al., 2018; Boycott et al., 2019). Amongst Mendelian disorders, developmental diseases with or without intellectual disability are highly heterogeneous and require extensive workup to establish a molecular diagnosis.

Exome sequencing (ES), the current gold-standard molecular test used for heterogeneous diseases with strong evidence of a genetic etiology, has a 30%–40% diagnostic yield, after negative cytogenetic tests including conventional karyotyping, sub-telomeric screening, array comparative genomic hybridization (array–CGH), and fragile X testing (Clark et al., 2018). Several limitations may explain a high percentage of undetected causative variants, such as poorly enriched exonic regions, pseudogenes, and non-coding variants within untranslated, intronic, and intergenic regions. In addition, inherited variants with incomplete penetrance and variable expressivity may further complicate the correct interpretation of their clinical significance (Bruel et al., 2020).

Short-read genome sequencing (srGS) has been considered a powerful second-tier approach for the molecular diagnosis of unsolved patients (Hartley et al., 2020). Several countries (e.g. the United States, Canada, UK, and France) have now invested in national human genome projects programs to identify the genetic causes of rare and common diseases as well as cancer and population genetics studies (Marshall et al., 2020; Sanlaville et al., 2021; Smedley et al., 2021).

As compared to ES, srGS discovers around two orders of magnitude more variants and requires the implementation of complementary approaches, including split-reads analysis to identify balanced structural variants (SVs), combined with read-depth analysis, to identify unbalanced structural variants below the detection limit of array-CGH (Caspar et al., 2018). However, it remains underused as a routine diagnostic laboratory approach in clinical settings due to the need for tailored bioinformatics pipelines, interpretation tools, and associated costs (Belkadi et al., 2015; Lelieveld et al., 2015; Meienberg et al., 2016). In the last 5 years, several publications have reported that, on average, GS improves diagnostic yield (10%–20%) compared to ES primarily by extending the analysis to non-ES-enriched coding and non-coding regions. Thus, srGS is effective in detecting variants within poorly-enriched exonic regions, and balanced and unbalanced structural variants not observed by the abovementioned techniques (Belkadi et al., 2015; Lelieveld et al., 2015). However, srGS cannot identify structural variants with breakpoints lying within repetitive DNA and GC-rich regions, hampering its ability to fully characterize complex structural variants (Bose et al., 2014). In this respect, low-depth (10–20X) long-read genome sequencing (lrGS) overcomes this problem by generating reads longer than 5 kilobases (Kb) in length, and up to 1–2 megabases (Mb) depending on the technology used, spanning repeated DNA or GC-rich regions, thus facilitating *de novo* genome assembly, SV calling and haplotype phasing using tailored bioinformatics pipelines (Sedlazeck et al., 2018; Chaisson et al., 2019; Amarasinghe et al., 2020; Mitsuhashi and Matsumoto, 2020). Optical genome mapping is a suitable alternative to lrGS. Using this approach SVs are identified by reconstructing high-resolution genomic maps obtained by detecting specific sequence motifs (fingerprints) along multiple single and extremely long fluorescently-labeled DNA fragments (ranging from 150 Kb up to 2 Mb).

Bionano Genomics optical mapping currently offers a resolution of up to 500 base pairs, thus facilitating first-line genome scaffolding and the detection of large-scale structural variations (Yuan et al., 2020).

However, this method requires additional investigations to achieve nucleotide resolution for the identification of breakpoints, and phase single nucleotide variants/small indels haplotypes.

Genetic information alone is sometimes insufficient to predict the impact of candidate variants on gene expression, transcription or RNA splicing. Interest in RNA sequencing is also increasing in the area of translational research to help identify or prioritize genetic variants (Cummings et al., 2020; Murdock et al., 2021; Yépez et al., 2022). In particular, RNA-seq analysis identifies aberrant splicing events, expression outliers and monoallelic expressions (Kremer et al., 2017; Peymani et al., 2022). Recent studies in clinically accessible sample tissues (i.e. blood, fibroblast or lymphoblastoid cell lines, bone marrow or skeletal muscle) reported a highly variable diagnostic yield of RNA-seq, in combination with ES or GS, between 7.5%–36% in defined cohorts of patients presenting primarily with neuromuscular, mitochondrial or metabolic diseases (Cummings et al., 2017; Kremer et al., 2017; Kremer et al., 2018; Frésard et al., 2019; Gonorazky et al., 2019; Hamanaka et al., 2019; Lee et al., 2020; Rentas et al., 2020; Stenton and Prokisch, 2020; Murdock et al., 2021; Yépez et al., 2022). Interestingly, clinically accessible samples are heterogeneous in terms of gene and transcript expression (GTEx - https://gtexportal.org/home/), (Lonsdale et al., 2013), indicating that their relevance for RNA studies in Mendelian disorders may not be the same, meaning their selection could impact the diagnostic outcome of RNA-seq testing (Rentas et al., 2020; Murdock et al., 2021). Although the interest in using GS, long-read sequencing, optical mapping and RNA-seq for establishing diagnoses in unsolved rare-disease patients, no standard consensus exists regarding the order of applying these approaches after negative or inconclusive routine results in patients with a typical clinical diagnosis.

To address this issue, we used 15 molecularly unexplained individuals diagnosed with an OMIM syndrome. Some patients presented with a recognizable syndrome (i.e., a genetic condition associated with distinctive features, cardinal symptoms, and signs), whose pathogenic variants could be expected to be identified within a relatively short list of well-known OMIM morbid genes. Clinical diagnostic indications of recognizable syndromes are a valuable aid in selecting the most cost-effective molecular approach to identify causative genetic variants. Depending on the possible genetic heterogeneity associated with the investigated disease, several first-line genetic tests, including targeted Sanger sequencing, gene panel testing or ES/GS, can be used to establish a molecular diagnosis before turning to more expensive pan-genomic analyses. Standard approaches, however, may sometimes miss causative variants. The individuals had been previously tested *via* gold standard diagnostic approaches including Sanger sequencing, gene panel sequencing, ES or array-CGH/SNP-array. Individuals had been clinically diagnosed with: 1) an autosomal recessive disease with a single known pathogenic variant identified, 2) an X-linked recessive disease or 3) a dominant disease. We performed srGS followed by RNA sequencing in clinically accessible samples selected based on the expression pattern of the expected causal genes, in cases with negative or inconclusive GS results. Optical mapping or long-read sequencing (lrGS) were used to resolve structural variants unable to be characterized by srGS alone.

## 2 Materials and methods

### 2.1 Individuals

The French AnDDI-rares network recruited affected individuals from eight investigating centers in Dijon, Rouen, Nantes, Rennes, Angers, Strasbourg, Reims in France, and Liège in Belgium. We obtained written informed consent for research purposes for the testing from all subjects or their legal representatives. A clinical description of all individuals is available in the Supplementary Data section.

### 2.2 DNA extraction, quality control and short-read genome sequencing and analysis

DNA was extracted from blood collected in EDTA tubes. We incubated 3–5 mL of whole blood for 10 min in RBC lysis buffer (Qiagen GmbH, Hilden, Germany) and then centrifuged it for 2 min at 2000 rpm to pellet white blood cells. The pellet was resuspended in 180 µL of residual supernatant and 20 µL of RNAse A (Qiagen GmbH, Hilden, Germany). DNA was then purified using the QiAamp DNA Blood mini kit on a QiaCube extraction device following the standard protocol.

DNA was quantified using the Qubit dsDNA HS Assay (Life Technologies, CA, United States) and qualified *via* gel electrophoresis. DNA purity was checked by evaluating the absorbance ratio A260/A280, to evaluate protein contamination, and the A260/A230 ratio, to evaluate organic solvent contamination, using a Multiskan Go device (Thermo Scientific, Waltham, MA, United States).

A minimum of 4 µg of DNA was needed per sample for quality control purposes before sequencing at the CNRGH platform and to potentially prepare a second library in the event of technical problems. The center was asked to provide another sample in the event of insufficient DNA quantity or quality.

Standard methods (described in Supplementary Methods) were used for data extraction. Genomic DNA libraries were prepared in accordance with the TruSeq DNA PCR-free protocol (Illumina, CA, United States). A minimum of 1 µg of genomic DNA was sheared by sonication and then purified. Oligonucleotide adaptors to sequence both ends were ligated on end-repaired fragments and then purified. DNA libraries were barcoded (indexed) and then multiplexed. GS was performed at the Centre National de Recherche en Génomique Humaine (CNRGH, CEA) using the Illumina NovaSeq6000 platform (Illumina, CA, United States), generating 150 base pairs paired-end reads. Data sequencing was required to meet minimum quality standards, with an average of over ×35 depth of coverage and over 97% of the genome covered by at least 10 reads.

Variants were identified using a computational platform of the FHU Translad, hosted by the University of Burgundy Computing Cluster (CCuB). Raw data quality was evaluated by FastQC software (v0.11.4—see web resources). Reads were aligned to the GRCh37/hg19 human genome reference sequence using the Burrows-Wheeler Aligner (v0.7.15) (Li and Durbin, 2009). Aligned read data underwent the following steps: (a) duplicate paired-end reads were marked by Picard software (v2.4.1—see web resources), and (b) base quality score was recalibrated using the Genome Analysis Toolkit (GATK v3.8) Base recalibrator (DePristo et al., 2011). Using GATK Haplotype Caller, Single Nucleotide Variants with a quality score >30 and an alignment quality score >20 were annotated with SNPEff (v4.3) (Cingolani et al., 2012). Rare variants were identified by focusing on non-synonymous changes at a frequency of less than 1% in the gnomAD database. Copy Number Variants (CNV) were detected using two approaches based on read-depth analysis using Control-FREEC (v11.4) (Boeva et al., 2012) and based on read-pair anomalies combined with split-read detection using Lumpy (v0.2.12) (Layer et al., 2014). The resulting CNV and SV were annotated using in-house python scripts and filtered in terms of their frequency in public databases (DGV, ISCA, DDD).

### 2.3 High molecular weight (HMW) DNA extraction, quality control, and long-read genome sequencing and analysis

HMW DNA extractions and sequencing were performed by the Centre National de Recherche en Génomique Humaine (CNRGH, CEA). DNA was extracted from frozen PBMCs or fibroblasts (2 to 3 million cells) using Circulomics Nanobind CBB DNA kits (Circulomics Inc., Baltimore, MD), following the standard protocol of the manufacturer. Extracted HMW gDNA was quantified using the Qubit dsDNA HS Assay (Life Technologies, CA, United States), and the quality of the HMW DNA assessed by migration of a small aliquot onto a Femto Pulse System (Agilent Technologies, Santa Clara, CA, United States). HMW DNA purity was verified by evaluating the absorbance ratios A260/A280 and A260/A230 using a NanoDropTM ND-1000 device (Thermo Scientific, Waltham, MA, United States). After gDNA fragmentation to a size of ∼20 kb using a Megaruptor®-2 (Diagenode) and size validation on a TapeStation 4200 (Agilent technologies, Santa Clara, CA, United States), libraries were prepared using an input of 1 µg 20 kb-fragmented-gDNA, following the Oxford Nanopore “Genomic DNA by Ligation” protocol, using the NEBNext^®^ Companion Module for OxfordNanopore Technologies^®^ LigationSequencing (cat #E7180S New England Biolabs) and the Oxford Nanopore Ligation Sequencing kit (cat # SQK-LSK109 or SQK-LSK110, ONT^®^). Each library was then sequenced on R9.4.1 flowcell using PromethION (ONT^®^). Base-calling on PromethION was performed using the Guppy version available at the time of processing the samples (Guppy 3.2.6 in 2020 and Guppy 4.3.4 in 2021).

We analyzed the rearrangements in the region targeted by the GS. Reads were aligned to the GRCh37/hg19 human genome reference sequence using the Minimap2 Aligner (v2.11) (39) and were extracted by samtools (version 1.9) (Li et al., 2009) for the chromosome of interest. In line with the recommendations of the official pipeline, chromosome-specific reads were aligned to the genome using Last (version 1080) (Frith et al., 2010). Rearrangements were identified by dnarrange (Mitsuhashi et al., 2020) and five controls. We retained rearrangements supported by at least three reads and grouped the reads with the same rearrangement into a consensus sequence using dnarrange-merge and lamassemble (Frith et al., 2021). Pictures of each consensus sequence were created using last-multiplot and last-dotplot (Frith et al., 2010). For genes of interest, we manually analyzed each group to reconstruct the complex rearrangement.

### 2.4 RNA sequencing and analysis

RNA was extracted from cultured cells or Blood PAXgene tubes. Cultured cells were washed in PBS and lysed in 1 mL Trizol (Ambion); 200 µL of chloroform was added, mixed, and incubated at room temperature (RT) for 3 min before centrifugation for 15 min at 4°C at 12 000 G. The RNA-containing supernatant was then retrieved, and 500 µL of isopropanol was added and mixed before being incubated for 15 min at room temperature and then centrifugated at ×12000 g, 4°C. The RNA-containing pellet was then washed twice with 75% ethanol and centrifugated for 5 min, ×7500 g, 4°C. Ethanol was then carefully eliminated, and the resulting dried-up pellet resuspended in 20 µL of nuclease-free water. For PAXgene Blood tubes, total RNA was extracted from whole blood collected in PAXgene tubes (Preanalytics GmbH, Hombrechtikon, Switzerland) using the PAXgene Blood RNA kit (Preanalytics GmbH, Hombrechtikon, Switzerland) automated on a QiaCube extraction device (Qiagen GmbH, Hilden, Germany) following the standard protocol. RNA was then quantified by absorbance measurement on a Nanodrop device. Quality was assessed by determining the RNA Integrity Number (RIN) on a bioanalyzer device (Agilent Technologies, Santa Clara, CA, United States). For RNA-seq, RNA with an RNA integrity Number (RIN) **≥**7 was used.

RNAseq sequencing was carried out at the CNRGH (CEA). After an RNA quality control on each sample (duplicate quantification on a NanoDrop™ 8000 spectrophotometer and RNA6000 Nano LabChip analysis on Bioanalyzer from Agilent), libraries were prepared using either the “TruSeq Total stranded RNA Library Prep Gold” from Illumina (individual 9085), or the “TruSeq Stranded mRNA Library Prep Kit” from Illumina (individual 13158). All libraries were prepared on an automated platform, using an input of 1 µg of total RNA, in accordance with the instructions of the manufacturer. Library quality was checked on a LabGx (Perkin Elmer) for profile analysis and quantification. Sample libraries were pooled before sequencing to reach the expected sequencing depth. Sequencing was performed on an Illumina HiSeq4000 as paired-end 100 bp reads, using dedicated Illumina sequencing reagents. Libraries were pooled using four samples per lane. Fastq files produced after RNA-seq sequencing were then processed using in-house CNRGH tools to assess the quality of raw and genomic-aligned nucleotides.

Aberrant splice events and expression outliers were identified using a computational platform of FHU Translad. Raw data quality was evaluated by FastQC software (v0.11.4.—see web resources). Reads were aligned to the GRCh37/hg19 human genome reference sequence using the STAR2 Aligner (v2.5.2b) (Dobin et al., 2013) with the 2-pass mapping method using the human RefSeq genome annotation (Build GCF_000001405.25[YD1]). Read counts were also collected using STAR2. Uniquely mapped reads were counted while overlapping only one gene. Outlier expressed genes were detected using two parallel methods: DESeq2 (v1.26.0) (Love et al., 2014) and Outrider (v1.4.2) (Brechtmann et al., 2018). After a normalization step, expression analysis was performed using the following analysis design: 1 versus all the analysis batch allowing computation of the expression variance on the whole cohort. A Z-score was computed, and filters applied to only retain genes with a z score above 3 or below −3. Aberrant splice events were detected using three parallel methods: rMATS (v4.0.2) (Shen et al., 2014), LeafCutter (v0.2.9) (Jenkinson et al., 2020), and a custom method derived from Cummings and collaborators (Cummings et al., 2017). We computed a Percent-Splice-In (PSI) value, indicating the proportion of the junction implied in a splice event, using rMATS. LeafCutter applies an intron-centered analysis using a clustering method. A Z-score was computed for both methods, applying the same filters as those used for expression. The custom method considered each splice junction as a rare variant and applied a filter based on frequency in the cohort to ensure only rare events were selected.

### 2.5 Ultra-high molecular weight genomic DNA extraction, optical mapping and data interpretation

Ultra-high molecular weight (UHMW) genomic DNA (gDNA) was extracted from 1.5 million white blood cells or fresh cultured fibroblast cells *via* the SP Blood & Cell Culture DNA Isolation Kit (ref: 80030, Bionano Genomics) according to the Bionano Prep SP frozen human blood DNA isolation protocol or the Bionano Prep SP fresh cells DNA isolation protocol. After quantification, 750 ng of UHMW gDNA was labeled *via* a DLS DNA Labeling Kit (ref: 80005, Bionano Genomics) according to the Bionano prep DLS protocol. Labeled UHMW gDNA was then analyzed on a Bionano Saphyr instrument with a target output of 500 Gbp. Raw data from Saphyr were directly exported in a BNX molecule-information file format. The *de novo* assembly was performed by the Bionano Solve pipeline (v3.6.11162020). Short molecules (<120 kbp) or molecules with insufficient labels (<9) were filtered out. Molecules were then sorted using a numeric molecular identifier. Molecule-noise parameters (including missing or extra labels, sizing intervals, and stretch factors) were estimated during the auto-noise stage. The resulting corrected BNX file contained rescaled molecules, which were aligned with each other using a pairwise algorithm producing the basis of an overlap graph. A first assembly was created from the pairwise alignments by analyzing potential paths on the chart, resulting in a consensus map (CMAP file). The longest paths were selected by successive clean-up steps. RefAligner software was used to further refine the draft consensus map in several stages by adjusting the labels and splitting map regions, where necessary, due to poor molecule coverage. The resulting refined consensus maps were then extended and merged iteratively to produce more contiguous maps. Maps were generated during a final refinement stage in which molecules were realigned to the maps. These maps were then modified when other molecules, typically those with alternative alleles, offered better results. The final consensus map was used to detect structural variants by comparing the map to a given reference, aligning the consensus map to the reference map. Results were produced in SMAP files convertible into VCF files. In a final stage, data was uploaded to the Bionano Access (v1.6.1) web server, to display the maps and detected variants.

## 3 Results

Fifteen molecularly unsolved individuals (12 males and 3 females) clinically diagnosed with OMIM-morbid syndromes previously tested using standard diagnostic methods were identified *via* the French AnDDI-rares network. Their clinical diagnosis had been established based on the presence of cardinal symptoms characteristic of a particular rare disease or by a reverse-phenotyping approach after identifying a single pathogenic variant in a gene linked to a recessive disease, compatible with the clinical presentation of the patient. In the eight individuals clinically diagnosed with an autosomal recessive disorder, first-line genetic investigations identified only one causative variant but failed to pick up a second hit. In the other individuals, negative results were obtained for first-line genetic in six individuals and identified a variant of unknown significance in one individual.

Diseases investigated included persistent Mullerian duct syndrome, type II (MIM 261550, autosomal recessive); developmental and epileptic encephalopathy 28 (MIM 616211, autosomal recessive); Cohen syndrome (MIM 216550, autosomal recessive); muscular dystrophy, limb-girdle, autosomal recessive 5 (MIM 253700, autosomal recessive); Alopecia-intellectual disability syndrome 4 (MIM 618840, autosomal recessive); muscular dystrophy, limb-girdle, autosomal recessive 23 (MIM 618138, autosomal recessive); Spermatogenic failure 5 (MIM 243060, autosomal recessive); primary ciliary dyskinesia-7 (MIM 611884, autosomal recessive); Simpson-Golabi-Behmel syndrome, type 1 (MIM 312870, X-linked recessive); Marfan syndrome (MIM 154700, autosomal dominant); Cowden syndrome (MIM 158350, autosomal dominant); tuberous sclerosis complex (MIM 613254, autosomal dominant); familial adenomatous polyposis 1 (MIM 175100, autosomal dominant); and KBG syndrome (MIM 148050, autosomal dominant) (Table 1, supplementary data).

**TABLE 1:** Cohort of 15 individuals with the different omic tests performed and their outcome. AD, autosomal dominant; AR, autosomal recessive; XLR, X-linked recessive; NP, not performed; sr-GS, short read genome sequencing; mRNA-seq, m RNA sequencing; OM, optical mapping; lr-GS, long read genome sequencing.

Individual	OMIM	Gene	Inheritance	Known variants				Newly identified variants				Technique(s) for diagnosis
				Array-CGH	Targeted sanger sequencing	Gene panel testing	Exome sequencing	Short read genome results	mRNA results	Optical mapping	Long read Genome	
1	Cowden syndrome, MIM 158350	*PTEN*	AD	—	NP	NP	—	NC_000010.10:g.89692904C>T	NP	NP	NP	sr-GS
2	Spermatogenic failure 5, MIM 243060	*AURKC*	AR	NP	NC_000019.9:g.57743438del	NP	NP	NC_000019.9:g.57746411C>G	NP	NP	NP	sr-GS
3	Tuberous sclerosis complex, MIM 613254	*TSC2*	AD	—	—	NP	NP	NC_000016.9:g.2121798del	NP	NP	NP	sr-GS
4	Simpson-Golabi-Behmel syndrome, type 1, MIM 312870	*GPC3*	XLR	—	—	NP	NP	NC_000023.10:g.133087134del	NP	NP	NP	sr-GS
5	Persistent Mullerian duct syndrome, type II, MIM 261550	*AMHR2*	AR	—	—	NC_000012.11: g.53823971_53823997del	NP	NC_000012.11:g.53823981C>T	NP	NP	NP	sr-GS
6	Marfan syndrome, MIM 154700	*FBN1*	AD	—	—	—	—	seq[GRCh37] t(9; 15)(p13.3;q21.1)dn	NP	NP	NP	sr-GS
								NC_000009.12:g.pter_33854979delins[NC_000015.9:g. 48757205_qter]				
								NC_000015.9:g. 48757198_qterdelins[NC_000009.12:g.33854965_pterinv]				
7	Muscular dystrophy, limb-girdle, autosomal recessive 5, MIM 253700	*SGCG*	AR	NP	NP	NP	NC_000013.10: g.23898591G	seq[GRCh37] del(chr13)(q12.12q12.12)	NP	NP	NP	sr-GS
								NC_000013.10: g.23889047_23896619del				
8	Primary ciliary dyskinesia-7 (CILD7), MIM 611884	*DNAH11*	AR	—	NP	NP	NC_000007.13: g.21747410del	seq[GRCh37] del(7)(p15.3p15.3)	NP	NP	NP	sr-GS
								NC_000007.13: g.21652528_21656729del				
9	Muscular dystrophy, limb-girdle, autosomal recessive 23, MIM 618138	*LAMA2*	AR	arr[GRCh37] 6q22.33(129380899_129451598)x1	—	—	—	seq[GRCh37] del(6) (q22.33q22.33)pat	NP	Complex structural variant including the partial duplication of *LAMA2* and *TMEM244* and the deletion of the intergenic region between them	Complex structural variant including the partial duplication of *LAMA2* and *TMEM244* and the deletion of the intergenic region between them	sr-GS and OM
								NC_000006.11: g.[129376140_129458794del				
								Complex structural variant inherited from the father				
10	Simpson-Golabi-Behmel syndrome, type 1, MIM 312870	*GPC3*	XLR	arr[GRCh37]	NP	NP	NP	seq[GRCh37] der(X) (q26.2,q24)mat	Aberrant *GPC3* expression	NP	NP	sr-GS and mRNA-seq
				Xq26.2(133 032 087_133 058 806)x1				NC_000023.10:g.(133030929_133031380)_(133079087_133079463)delins(118528009_118528409)_(118674690_118675082)				
				Xq24(118 531 987_118 673 787)x3								
11	Developmental and epileptic encephalopathy 28, MIM 616211	*WWOX*	AR	—	—	NP	NC_000016.9: g.78133710C>G	seq[GRCh37] der(16)del(16)(q23.1q23.1)inv(16)(q23.1q23.1)del(16)(q23.1q23.1)NC_000016.9: g.78179358_78219143delins78185355_78199419inv	Exon 5 skipping	NP	NP	sr-GS and mRNA-seq
12	Cohen syndrome, MIM 216550	*VPS13B*	AR	ND	NC_000008.10: g.100829801_100829802dup	ND	ND	NC_000008.10:g.100125810G>T	Creation of an acceptor splice site and exon retention	NP	NP	sr-GS and mRNA-seq
13	Alopecia-intellectual disability syndrome 4, MIM 618840	*LSS*	AR	NP	NC_000021.8:g.47614438G>A	NP	NP	seq[GRCh37] del(21) (q22.3q22.3)	Monoallelic expressionAberrant splicing	NP	Small deletion of 339 bp encompassing exon 1. and the intergenic region between *LSS* and *MCM3AP*	sr-GS, mRNA-seq and lrGS
								NC_000021.8:g.47648580_47648918del				
14	Familial Adenomatous Polyposis 1, MIM 175100	*APC*	AD	NP	—	—	—	—	—	NP	NP	—
15	KBG syndrome, MIM 148050	*ANKRD11*	AD	—	NP	—	—	—	—	NP	NP	—

### 3.1 Diagnosis established by GS

#### 3.1.1 Individual 1# *PTEN*

Individual 1 was a 30-year-old male, the third child of non-consanguineous French parents, presenting with a typical Cowden syndrome (Supplementary Figures S1A–C). Immunofluorescence confirmed an absence of PTEN expression in thyroid tissue. Genetic investigations consisting of array-CGH and ES with good coverage and depth on *PTEN* were negative. GS revealed a non-sense variant p.(Arg303*) in exon 6 of *PTEN* (Supplementary Figure S1D). Reanalysis of previous ES data revealed the presence of the variant on the Integrative Genomics Viewer but also showed that it had been filtered out by the bioinformatic pipeline of the service provider.

#### 3.1.2 Individual 2# *AURKC*

Individual 2 was a 34-year-old male from a family with seven children (six boys) with no noteworthy family medical history, presenting with typical Spermatogenic Failure 5. Genetic investigations consisting of *AURKC* Sanger sequencing identified a single frameshift variant p.(Leu49Trpfs*23) in exon 3 of *AURKC*. The variant was in gnomAD (v2.1.1) and classified as pathogenic in ClinVar. GS identified a second ClinVar pathogenic hit, i.e., a non-sense variant p.(Tyr248*) in exon 6 of *AURKC* (Supplementary Figures S1E–F)*.* Revaluation of *AURKC* Sanger sequencing data revealed the presence of a barely detectable peak, corresponding to the NM_01015878.1:c.744C>G variant identified by srGS. This false negative result was due to partially degraded sequencing primers on a single analysis batch.

#### 3.1.3 Individual 3# *TSC2*

Individual 3 was a 2-year-old girl, the first child of unaffected, non-consanguineous African parents. Tuberous sclerosis complex (TSC) was diagnosed prenatally. Prenatal and postnatal genetic investigations including screening for TSC in amniotic fluid and then blood and saliva samples using a specific panel were negative. GS analysis identified a frameshift variant p.(Gly654Alafs*44) in exon 19 of *TSC2* (Supplementary Figure S1G). Reanalysis of *TSC2* panel data revealed a bioinformatic pipeline anomaly that had filtered out this variant. In fact, it was artifactually detected in all patients from all batches but at an allelic frequency ranging between 0.7%–25% and associated with a sequencing strand bias.

#### 3.1.4 Individual 4# *GPC3*

Individual 4 was a 6-year-old boy, the oldest child of unaffected, non-consanguineous French parents, with a clinical diagnostic of Simpson-Golabi-Behmel syndrome and a familial history compatible with X-linked inheritance. *GPC3* Sanger sequencing was negative. GS revealed a hemizygous frameshift variant p.(Gln94Serfs*10) in exon 2 of *GPC3* (Supplementary Figures S1H, I). This variant was absent in gnomAD (v2.1.1). Familial segregation analysis revealed maternal inheritance. The variant was also identified in a hemizygous state in his affected brother. Revaluation of *GPC3* Sanger sequencing data revealed the presence of the variant that had been missed due to poor data quality.

#### 3.1.5 Individual 5# *AMHR2*

Individual 5 was a 37-year-old male, the third child of unaffected, non-consanguineous French parents, having been diagnosed with Persistent Müllerian duct syndrome (PMDS), and a normal karyotype (46, XY). Targeted mutational *AMHR2*-gene screening identified only one pathogenic variant, a maternally inherited recurrent 27-bp deletion in the kinase domain. Five *AMHR2* promoter sequencing and *AMHR2* southern blot performed to discover the second pathogenic allele produced negative results. Sanger sequencing of *AMH* (Anti-Mullerian hormone) was also negative. GS identified the second allele, which consisted of a missense variant p.(Thr447Ile) in exon 10 of *AMHR2* inherited from the father in the kinase domain within the interval of the first deletion (Supplementary Figures S1J, K). This variant was absent in gnomAD (v2.1.1). *In silico* prediction scores were in favor of its pathogenic effect.

#### 3.1.6 Individual 6# *FNB1*

Individual 6 was a 23-year-old male, the third child of unaffected, non-consanguineous French parents, with a typical clinical diagnosis of Marfan syndrome with ectopia lentis (Supplementary Figures S2A, B). The presence of ectopia lentis was highly suggestive of the implication of the *FBN1* gene since this clinical feature is not one of the other Marfan syndrome genes. Previous genetic investigations, consisting of Sanger sequencing and MLPA techniques of *FBN1*, panel genes of Marfan syndrome and related diseases, array-CGH, and ES solo were negative. GS revealed a *de novo* balanced translocation which disrupted exon 40 of *FBN1* (Supplementary Figure S2C). The translocation was confirmed by conventional karyotyping (Supplementary Figure S2D).

#### 3.1.7 Individual 7# *SGCG*

Individual 7 was a 33-year-old male, the third child of unaffected non-consanguineous French parents, with a clinical diagnosis of limb-girdle muscular dystrophy. Immunofluorescence on muscle biopsy showed a pattern consistent with a sarcoglycanopathy. Singleton ES identified only one heterozygous missense variant p.(Glu263Lys) in exon 8 of *SGCG* (Supplementary Figure S3A), previously described in the literature (DiCapua and Patwa, 2014; Al-Zaidy et al., 2015). ClinVar and HGMD databases have reported this variant as pathogenic. No additional single nucleotide, indels, or copy number variants were detected from ES data. GS confirmed the presence of the previous variant and identified a heterozygous intragenic deletion of about 6.5 kb encompassing exon 7 (124 bp) of *SGCG* and expected to produce an out-of-frame variant p.(Arg193Serfs*46) (Supplementary Figure S3B). This deletion was confirmed by qPCR, and the segregation analysis revealed that deletion was inherited from the mother (Supplementary Figure S3C – Supplemental material and methods).

#### 3.1.8 Individual 8# *DNAH11*

Individual 8 was the first child of unaffected, non-consanguineous French parents. Gestational diabetes occurred during pregnancy requiring only health and dietary guidance. At 23 weeks of gestation (WG) + 2 days, ultrasound scans revealed complex cardiac malformation. After counseling, the couple opted for termination of pregnancy at 24 W G + 5. The fetal autopsy found situs abnormalities suggesting Ivemark syndrome type 1. Prenatal array-CGH was negative. Trio ES showed a heterozygous frameshift variant p.(Phe2214Trpfs*35) in *DNAH11* (Supplementary Figure S3D), inherited from the father*.* No additional single nucleotide, indels, or copy number variants were detected from ES data. GS identified a heterozygous intragenic deletion of about 4.2 kb encompassing exons 21 and 22 of *DNAH11* (Supplementary Figure S3E).

#### 3.1.9 Individual 9# *LAMA2*

**FIGURE 1:**
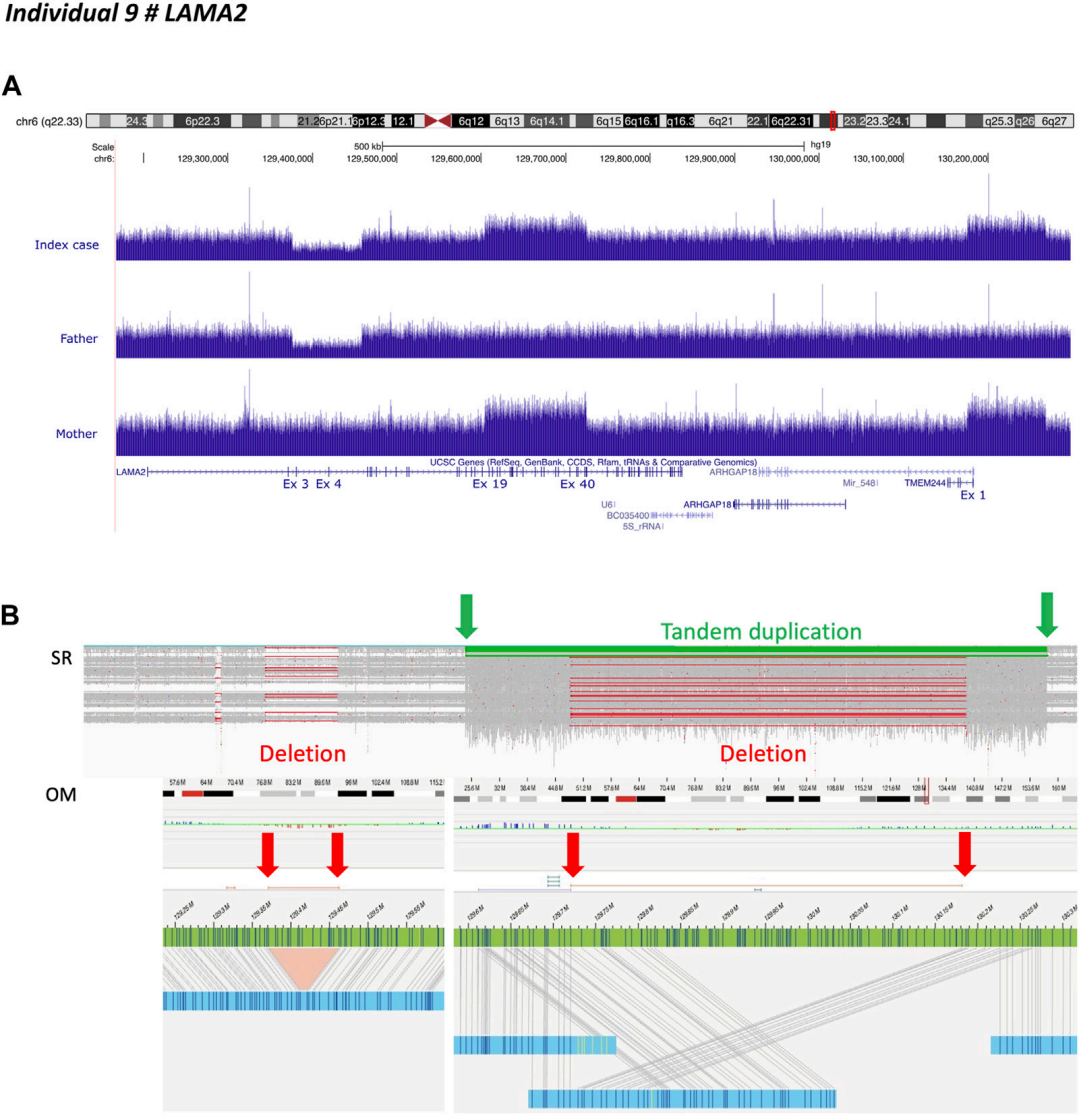
Individual 9—Muscular dystrophy, limb-girdle, autosomal recessive 23, MIM 618138. **(A)** Ideogram showing chromosome 6 and *LAMA2* localization. UCSC genome browser snapshot with visualization of *LAMA2* sequencing depth with an intragenic deletion encompassing exons 3 and 4 inherited from heterozygous father and intragenic duplications affecting exons 19 through 39 of *LAMA2* and the first exon of *TMEM244*, inherited from heterozygous mother. **(B)** Short-read sequencing and optical genomic mapping (OM) results demonstrated that *LAMA2* and *TMEM244* duplications belonged to the same complex event, including a partial deletion of the intergenic region within the duplicated fragment (i.e. copy neutral region). The insertion breakpoint is indicated by the green arrow on the left.

Individual 9 was a 14-month-old boy, the first child of unaffected, non-consanguineous French parents, presenting with neonatal hypotonia, and joint contractures compatible with congenital muscular dystrophy. Previous genetic investigations consisting of array-CGH had shown paternal inherited deletion of exons 3 and 4 of *LAMA2*. SrGS confirmed the presence of the 82 kb heterozygous intragenic deletion encompassing exons 3 and 4 of *LAMA2*. It also identified a heterozygous complex structural variant not resolvable by srGS alone, containing a 120 kb intragenic duplication encompassing exons 19 to 39 of *LAMA2* followed by a 92 kb duplication including the first exon of *TMEM244* fused into a single segment lacking the intergenic region, indicating they were from a single duplication/deletion event (Figures 1A, B). GS revealed the maternal inheritance of this complex structural variant (Figure 1A).

We used long-read genome sequencing (Oxford Nanopore) and optical genome mapping (Saphyr—Bionano Genomics) to resolve this complex structural variant. They confirmed the results obtained by srGS. However, the results were discordant regarding the position of the duplicated region, the latter indicating *LAMA2* while the former downstream *TMEM244* (Figure 1B and Supplementary Figure S4A). These conflicting results were due to the length of genomic fragments. As optical genome mapping analyzes assembled genomic fragments up to the megabase scale, it outperforms long-read sequencing. Thus revealing that the breakpoint was located near label position NC_000006.11:g.129611978 (Figure 1B). The insertion contained a chimeric fragment composed of partial duplication of *LAMA2* (NC_000006.11:g.129611978_129819640), a breakpoint between label positions NC_000006.11:g.129720651 and NC_000006.11:g.129733425, and a partial duplication of *TMEM244* and its downstream region (NC_000006.11:g.130181530_130263816) (Figure 1B). A reevaluation of GS data defined breakpoint at position NC_000006.11:g.129604517 in intron 18. The inserted fragment interrupted LAMA2 at intron 39, resulting in a loss-of-function allele (Figure 1B).

### 3.2 Diagnosis established by GS combined with RNA explorations

#### 3.2.1 Individual 10# *GPC3*

**FIGURE 2:**
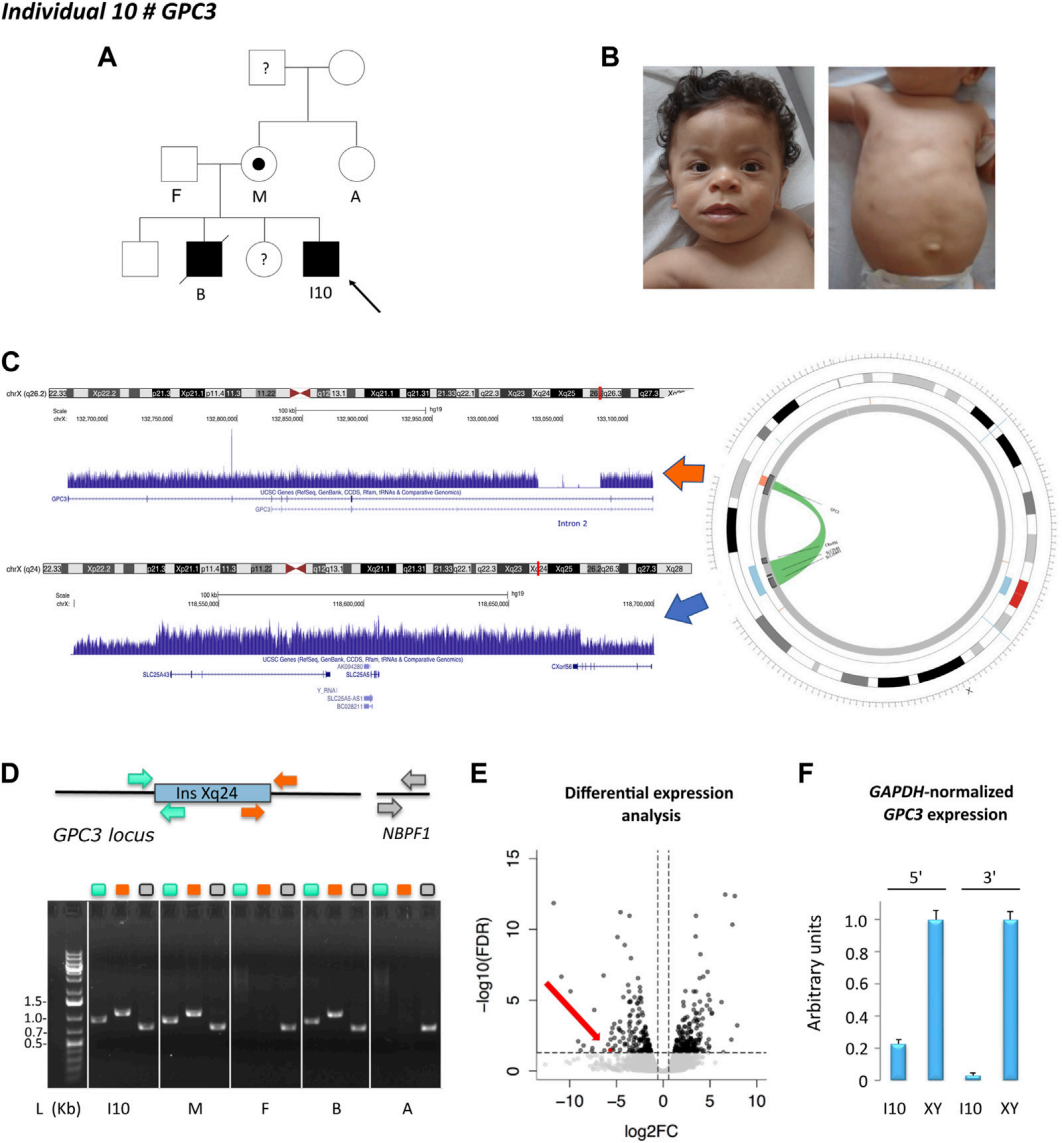
Individual 10—Simpson-Golabi-Behmel syndrome, type 1, MIM 312870. **(A)** Pedigree of individual 10. The affected status is indicated by filled symbols; the carrier is indicated by a dot symbol. **(B)** Dysmorphic features include telecanthus, palpebral edema, wide mouth, macroglossia, thin upper lip vermilion, smooth philtrum, supernumerary nipple, and umbilical hernia. **(C)** X chromosome ideogram and *GPC3* and Xq24 loci. Read depth tracks showing a deletion of 30 Kb in intron 2 of *GPC3* and an Xq24 duplication encompassing *SLC25A43*, *SLC25A5*, and the first exon of *CXorf56*. Split-read analysis indicated that these copy number variants belonged to the same event involving the insertion of the Xq24 region within the deleted intronic region of *GPC3,* visualized in the circos plot. **(D)** Schematic representation of the *Xq24* insertion in the *GPC3* locus and the control region *NBPF1*. The insertion-deletion event was validated by PCR using two couples of primers as indicated in the color scheme. Gel lanes are labeled according to the primer scheme. L: ladder, I10: index case, M: mother, F: father, B: brother, A: aunt. **(E)** RNA-sequencing differential expression analysis in fibroblast cell line showing loss of *GPC3* expression (red dot. and arrow) in individual 10 as compared to a cell line obtained from a healthy (unaffected) male. **(F)** qPCR results at 5′ and 3′. Ends of GPC3 cDNA. The results were normalized according to GAPDH expression and represented as relative expression, according to a healthy (unaffected) male (XY).

Individual 10 was a 5-year-old boy, the fourth child of unaffected, non-consanguineous Tunisian parents, diagnosed with Simpson-Golabi-Behmel syndrome with a family history compatible with X-linked inheritance (Figures 2A, B). Genetic investigations consisting of array-CGH and overgrowth syndrome panel showed a duplication of 140 kb in Xq24, encompassing *SLC25A43* and the 5′ terminal of *CXorf56*, considered as likely benign as duplications of these regions are found in the control population. However, in view of the clinical indication, a targeted inspection of the *GPC3* locus revealed the presence of a single deviated probe suggesting a small deletion in intron 2. ES analysis confirmed that the exonic regions were not affected by the deletion. GS showed that both structural variants were part of the same event involving the insertion of the Xq24 region within the deleted region in the second intron of *GPC3*, mediated by SINE elements located at both ends of the two regions (Figure 2C). These results were corroborated by PCR analysis, using two couples of primers designed to selectively amplify the insertion-deletion event within the *GPC3* locus and encompassing the two genomic breakpoints (Figure 2D). Segregation analysis revealed that the insertion-deletion event was present in the elder affected brother and the mildly affected mother but was absent in the healthy maternal grandmother and aunt. The healthy maternal grandfather was not available for DNA testing (Figure 2D).

RNA-sequencing in a fibroblast cell line derived from the affected individual compared to control male lines showed a loss of *GPC3* expression (Figure 2E), also corroborated by qPCR on cDNA (Figure 2F). RNA-sequencing also indicated that no alternative or fusion transcript originated from this locus.

#### 3.2.2 Individual 11# *WWOX*

Individual 11 was a 7-year-old girl, the second child of unaffected, non-consanguineous French parents, presenting with epileptic encephalopathy with limb spasticity, ataxia, dysmetria, movement disorders, and stereotypy. Previous genetic investigations consisting of array-CGH, screening for Angelman syndrome (DNA methylation analysis and *UBE3A* sequence analysis), and mitochondrial DNA sequencing were negative. Trio ES showed a missense variant inherited from the mother p.(Thr12Arg) in exon 1 of *WWOX* (Supplementary Figures S5A, B)*,* which was absent in gnomAD with *in silico* prediction scores in favor of its pathogenicity. GS identified the second event, which consisted of a complex rearrangement with two intronic deletions (introns 4 and 5) associated with an inversion of exon 5 (Supplementary Figures S5C, D). Sanger sequencing analysis of PCR-generated amplicons encompassing the breakpoints revealed a 19 bp insertion in the chimeric fragment involving the 3′-end junction (i.e., fragment 2 Supplementary Figure S5D – Supplemental material and methods). An PCR analysis of WWOX transcript, using primers encompassing exons 4 through 6, obtained from blood, revealed the presence of a shorter amplicon isoform (186 bp) in addition to the expected 293 bp band in individual 11 and her father, but not in the mother (Supplementary Figure S5E). Amplicon-sequencing analysis of the 186 bp gel-purified PCR product demonstrated that the structural variant caused exon 5 skipping in individual 11 and also in her father (Supplementary Figures S5E, F – Supplemental material and methods). Western blotting revealed WWOX loss in a fibroblast cell line obtained from a skin biopsy of Individual 11 compared with a sample from a healthy individual, confirming the pathogenic effect of the two identified variants (Supplementary Figure S5G – Supplemental material and methods).

#### 3.2.3 Individual 12# *VPS13B*

**FIGURE 3:**
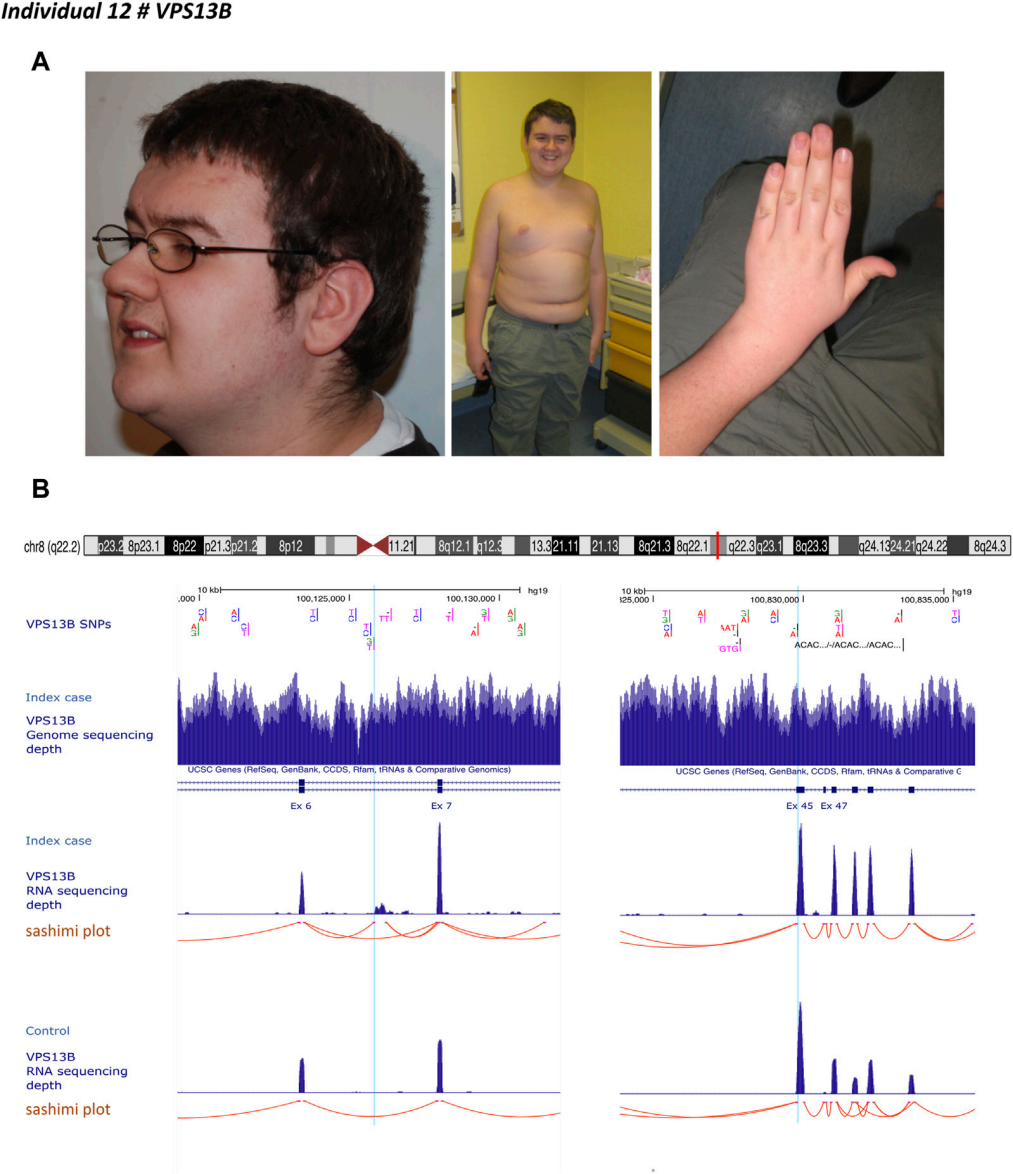
Individual 12—Cohen syndrome, MIM 216550. **(A)** Dysmorphic features include down-slanting palpebral fissures, midface retrusion, short philtrum, open mouth appearance, high palate, micrognathia, truncal obesity, narrow hand with tapered fingers, and joint hyperextensibility. **(B)** Ideogram showing chromosome 8 and *VPS13B* localization. UCSC genome browser snapshot with visualization of *VPS13B* single nucleotide polymorphisms (SNP), *VPS13B* sequencing depth, *VPS13B* RNA-sequencing depth and splicing analysis (sashimi plot) in individual 12 and a healthy individual (control). Left panel, visualization of the deep intronic heterozygous variant (NC_000008.10:g.100125810G > T) in intron 6, indicated by a light blue line and corresponding to the beginning of the cryptic exon. Red lines indicate spice events. This variant resulted in the creation of an acceptor splice site visualized on the splice analysis tract. The right panel shows the variant inherited from the mother indicated by the light blue line.

Individual 12 was a 30-year-old male, the first child of unaffected, non-consanguineous French parents whose autosomal recessive Cohen syndrome has been diagnosed due to the association of retinitis pigmentosa, ID, and specific facial features (Figure 3A). Targeted mutational screening of *VPS13B* had identified a maternally inherited pathogenic heterozygous frameshift variant p.(Tyr2711*). GS analysis was negative for structural variants accounting for the phenotype of the patient. RNA-seq analysis performed in patient-derived fibroblasts revealed the presence of a cryptic 422 bp exon located between exons 6 and 7. Reevaluation of GS data showed a deep intronic heterozygous variant (NC_000008.10:g.100125810G>T, LRG_3514t1:c.763-2118G>T) in intron 6 of *VPS13B*, inherited from the father and predicted to create an acceptor splice site (SpliceAI acceptor gain 0.28 at 3 base pairs downstream to it). A cryptic splice donor site was predicted at position NC_000008.10:g.100126234 (Figure 3B). The variant was absent in gnomAD (v2.1.1). The cryptic exon altered the open reading frame by creating a premature stop codon p.(Ile255Thrfs*7).

#### 3.2.4 Individual 13# *LSS*

**FIGURE 4:**
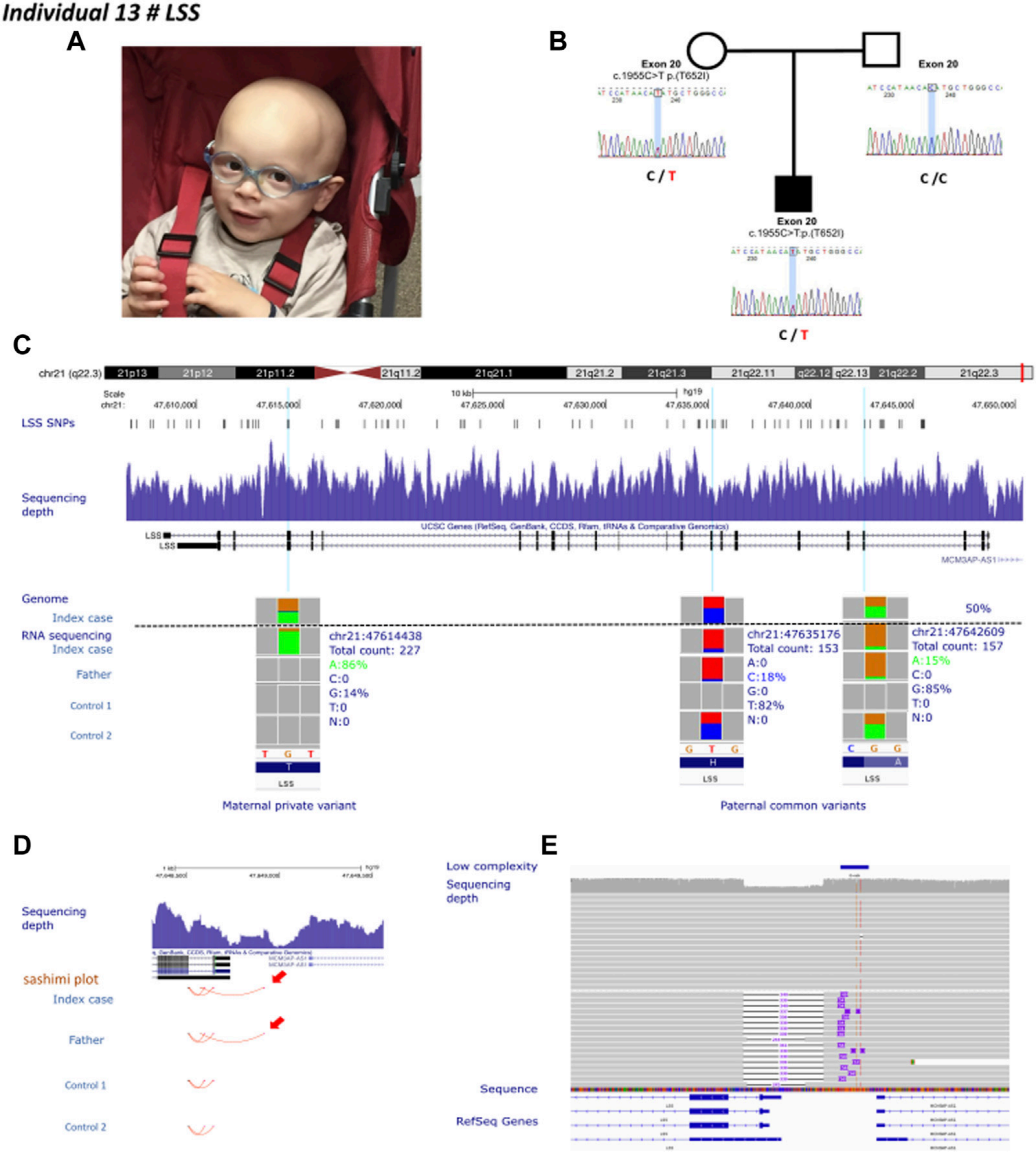
Individual 13—Alopecia-intellectual disability syndrome 4, MIM 618840. **(A)** Dysmorphic features showing alopecia universalis. **(B)** Pedigrees of individual 13. The known maternally inherited *LSS* pathogenic variant was Sanger sequenced. *LSS* pathogenic variants are highlighted in red. **(C)** Ideogram showing chromosome 21 and *LSS* localization. UCSC genome browser snapshot with visualization of LSS SNPs, sequencing depth, and genome and RNA-sequencing allelic balance from IGV (integrative genomics viewer) of three variants, one private maternal variant and two common paternal variants in individual 13, his father and two healthy controls, confirming monoallelic expression of the maternal allele in individual 13. **(D)** Visualization of the promoter region of *LSS*. Red arrows show aberrant splicing within the intron 1 of *LSS* and the intergenic region between *LSS* and *MCM3AP* in individual 13 and his father. **(E)** IGV visualization of long-read Oxford Nanopore genome sequencing showing the sequencing depth and a heterozygous deletion affecting the promoter region of LSS.

Individual 13 was a 4-year-old boy, the second child of non-consanguineous healthy parents, presenting with a clinical diagnosis of Alopecia-intellectual disability syndrome 4 (Figure 4A). His case was published by Besnard et al. (2019). Sanger sequencing identified only one maternally inherited *LSS* missense variant (c.1955C>T; p.Thr652Ile) in exon 20 and absent in gnomAD (v2.1.1). Linkage analysis using single nucleotide polymorphisms of *LSS* showed that individual 13 and his unaffected sister had inherited the same paternal allele but a different maternal allele. The authors also observed an unbalanced expression in favor of the maternal allele. These results were confirmed by RT-PCR in fibroblast from individual 13 and his father. The cause of this allelic imbalance was unexplained. We performed GS in individual 13, and RNA-sequencing in individual 13 and his father (Figure 4B). GS did not reveal any apparent structural variant (Figure 4C). Transcriptome analysis identified the second hit, i.e., an aberrant splice event involving exon 1 and promoter region of *LSS* (Figure 4D). RNA-seq data also confirmed an altered allelic expression of the maternal allele in individual 13 and a monoallelic expression of *LSS* in the father (Figure 4C). Reevaluation of GS at the promoter region indicated an unevenly distributed sequencing depth and the potential presence of an ill-defined SV. The predicted SV was too small to be characterized by optical mapping (i.e., below 500 bp). Oxford Nanopore long-read sequencing was therefore used to resolve this region. A long-read data analysis confirmed the presence of a small deletion of 339 bp encompassing exon 1 and the intergenic region between *LSS* and *MCM3AP* (Figure 4E).

### 3.3 Negative and inconclusive results

#### 3.3.1 Individual 14# suspected *APC*

Individual 14 was a 34-year-old female, the third child of unaffected, non-consanguineous French parents. A colonoscopy performed at the age of 28 due to abdominal pain revealed multiple colonic and rectal polyposis and adenocarcinoma of the transverse colon. The presence of polyposis at this early age suggested a need for genetic testing for a predisposition to polyposis. Targeted mutational screening of the *APC* and *MYH* was performed on blood samples and digestive tract biopsies; gene panels for familial adenomatous polyposis and ES were negative. Replication error (RER) phenotyping of the tumor revealed a microsatellite stable (MSS) phenotype and, in terms of immunohistochemistry, persistent expression of hMLH1, hMSH2, hMSH6, and PMS2 proteins was observed. GS and RNA-sequencing in fibroblasts detected no apparent anomalies, particularly no candidate variant in *APC*.

#### 3.3.2 Individual 15# suspected *ANKRD11*

Individual 15 was a 22-year-old male, the first child of a single French mother, presenting with a clinical diagnosis of KBG syndrome. Previous genetic investigations, including array-CGH, intellectual disability panel including *ANKRD11*, and solo ES, produced normal results. GS and RNA-sequencing detected no obvious anomalies. However, the srGS reanalysis identified a variant of unknown clinical significance in *OPHN1* (MIM 300486) (NM_002547.2:c.701A>C, p.Asn234Thr) predicted to alter RNA splicing (SPiP 85.91%). OPHN1 expression was not detectable in blood by RNA-seq, and we were unable to investigate the functional impact of this variant on the messenger RNA.

## 4 Discussion

Our study pinpoints the importance of phenotype-driven genomic investigations for identifying causative variants in rare diseases. From a clinical perspective, it is seminal to describe clinical terms with a standardized vocabulary (Human Phenotype Ontology—HPO terms), family history, and mode of inheritance. The goal of using common phenotypic semantics is to cover all phenotypic abnormalities commonly encountered in human monogenic diseases (Köhler et al., 2019). Deep phenotyping allows the characterization of robust diagnostic hypotheses. Different diagnostic approaches should be used if the phenotype is convincing, despite negative first-line genetic testing, or in the presence of only a single heterozygous pathogenic variant in the case of an autosomal recessive mode of inheritance (Frésard and Montgomery, 2018). Here, we described a combined omics strategy to solve 15 unrelated individuals diagnosed with rare Mendelian diseases for which first-line genetic investigations failed to establish a molecular diagnosis. We applied in a stepwise manner GS and mRNA-seq on patient-derived samples, optical genome mapping and long-read sequencing. Overall, these approaches resulted in a molecular diagnosis in 87% of the cohort (13/15 individuals). GS alone identified five causal SNVs/indels, four of which had been missed by first-line targeted tests and four structural variants. The mRNA-seq analysis confirmed the deleterious outcome of two complex rearrangements detected by GS and discovered one structural variant and one deep intronic SNV not previously identified by GS alone.

The failure to identify causal variants in known disease-causing genes by first-line genetic investigations, such as targeted sequencing, gene panel or exome sequencing, can be explained in numerous ways. It can be missed as interpreting the data can be challenging, hence re-analyzing the available data is key before carrying out further investigations. Technical limitations include biases in sequencing data (e.g., reduced coverage/sequencing depth, locus-specific features such as GC-extreme regions, homopolymeric repeats), or bioinformatics issues (Ross et al., 2013). For example, the diagnosis of individuals 1 and 3 allowed a correction of the exome and the panel bioinformatic pipelines of the respective centers. The causal variant can be considered of unknown significance, as the mechanism responsible for the disease may not be obvious (Richards et al., 2015). Functional assays and integration of genomics with other omics data (e.g., transcriptomics, proteomics, epigenomics, or metabolomics where appropriate) will thus enhance genotype-phenotype relationships for variant classification. In our study, a result was obtained for individual 11 using this integrated strategy. Structural variants cannot be adequately picked up, as seen in individuals 7, 8, 9, and 11. Detecting somatic mosaicism is also challenging as the distribution of causative variants is non-random and often absent or in a low level in blood samples. In addition, rare variants located in non-coding regions, such as deep intronic or regulatory variants, may need additional lines of evidence to demonstrate their implication in human disease, as shown in individuals 12 and 13 (Short et al., 2018; Bodle et al., 2021; Bryen et al., 2021).

In our experience, genomics and integrating analyses, such as pan-genomic or targeted transcriptomics approaches, should be used as a first resort to increase the diagnostic yield in negative patients analyzed by first-line genetic tests. GS can identify genomic variants in the coding and non-coding regions, such as indels, CNVs, and chromosomal rearrangements (Gilissen et al., 2014; Belkadi et al., 2015; Boycott et al., 2019). Structural variants are probably more frequently involved in Mendelian inheritance than expected, as observed in our cohort and reported elsewhere (Sanchis-Juan et al., 2018). It remains a bioinformatic challenge to detect and characterize such variants (Mahmoud et al., 2019; Kobren et al., 2021). Re-analyzing the available data using the latest available reference human genome combined with various tools for detecting CNVs may facilitate the identification of variants missed during previous analyses. Long-read sequencing technology and optical mapping approaches may now improve their detection (Chan et al., 2018; Chaisson et al., 2019; Logsdon et al., 2020).

Interpreting coding variants of unknown significance or non-coding variants is also challenging, advocating the need for the integration of transcriptomic data for variant reclassification. Transcriptomics can identify skewed allelic RNA expression, gene expression outliers, splicing aberrations, and gene fusions. So far, several computational approaches have been developed, for transcript abundance or differential splicing analyses (Cummings et al., 2020; Mehmood et al., 2020; Shahjaman et al., 2020). Recent studies have estimated that the diagnostic yield associated with RNA-seq, in combination with ES or GS, ranges from 7.5%–35% (Kremer et al., 2018; 2017; Cummings et al., 2017; Frésard et al., 2019; Gonorazky et al., 2019; Hamanaka et al., 2019; Lee et al., 2020; Stenton and Prokisch, 2020). Murdock et al. (2021) showed that a transcriptome-directed approach resulted in a diagnostic rate of 12% across their entire cohort, or 17%, after excluding cases solved on ES/GS alone. The diagnostic rate associated with RNA-seq is higher in cohorts with well-defined diseases, with an appropriate clinically accessible sample suitable for modeling candidate gene expression and splicing. In fact, tissue and isoform expression are essential factors to consider for RNA-seq analysis. In their cohort of 115 undiagnosed patients with diverse phenotypes, Murdock et al. (2021) showed that RNA-seq derived from fibroblast cell lines exhibited higher and less variable gene expression in clinically-relevant genes. Clinically accessible tissues such as blood samples, skin and muscle biopsies, as well as cell cultures (e.g., Epstein–Barr-transformed lymphocytes, fibroblast cell lines) are sometimes inadequate to model genes prevalently expressed in the central nervous system (Aicher et al., 2020). Reprogrammed neurons or astrocytes obtained from induced pluripotent cell lines should be considered when modeling neural-specific genes (Bronstein et al., 2020).

**FIGURE 5:**
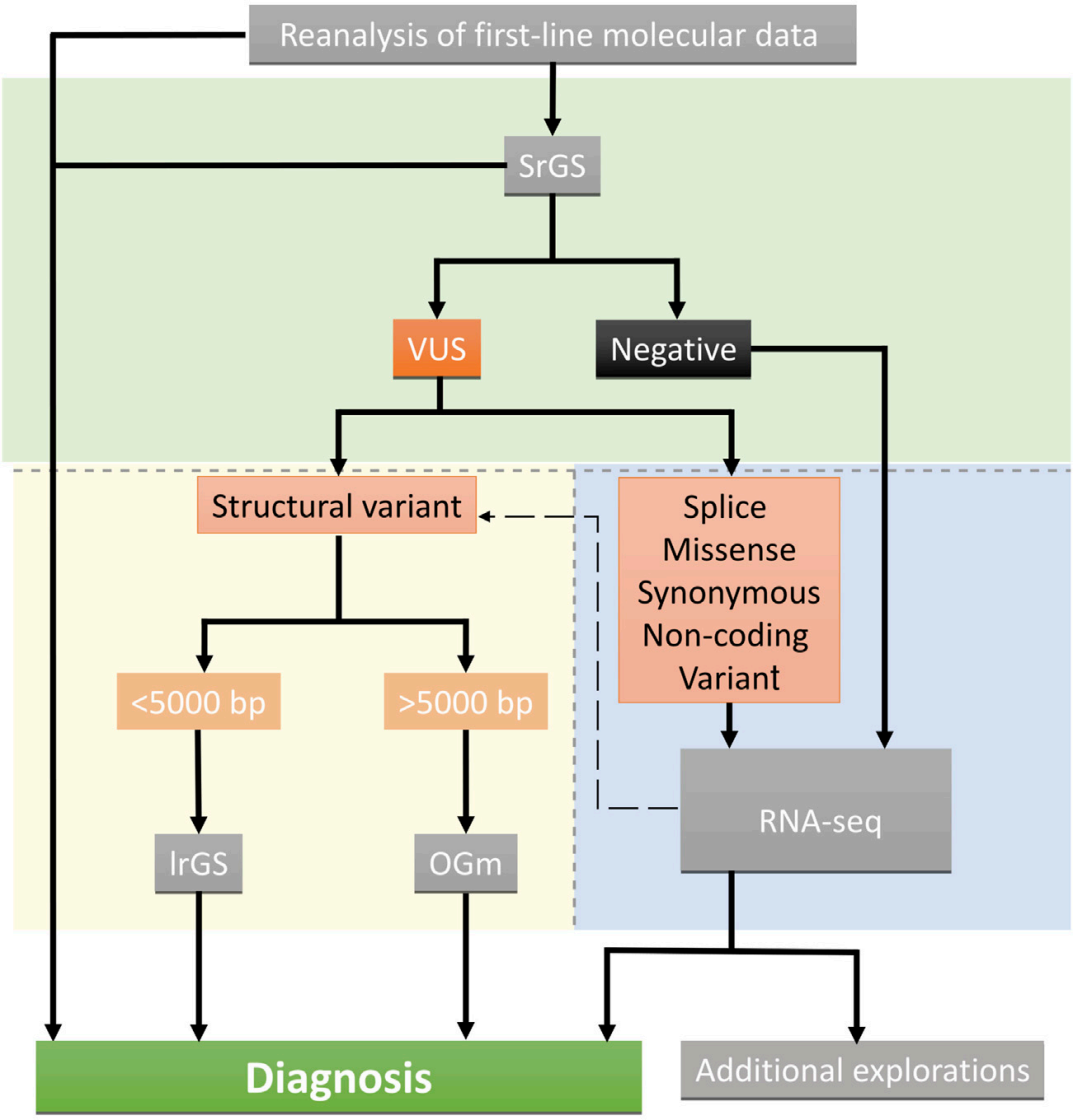
Suggested decision tree indicating the omic strategy implemented after inconclusive or negative results. Green, blue, and yellow backgrounds indicate short-read genome technologies, and transcriptome and long-read optical mapping technologies, respectively. Technics are shown in grey boxes.

Overall, we showed that typical clinical diagnosis of rare Mendelian diseases with negative or inconclusive first-line investigations could be largely resolved by the sequential use of srGS and mRNA-seq. With optical mapping and long-read genome sequencing we resolved one complex structural variant and one small deletion, identified either by srGS alone or in combination with RNA-seq, respectively. Overall, this approach allowed us to identify a molecular diagnosis in 13 out of 15 patients (87%). Our findings suggest that srGS should be used to guide the next approach in case of negative or inconclusive results in a multi-step analysis. RNA-seq should be used as a first-line analysis after negative srGS results or to reclassify variants of unknown significance, with a suspected splicing effect, in suitable clinically accessible samples. Structural variants not resolvable by srGS alone may benefit from optical mapping and/or long-read genome sequencing, selected according to the length, composition, and complexity of the structural variant (Figure 5). Longer term, national genomic medicine programs may benefit from implementing this type of analytical approach to reduce diagnostic deadlocks, even in heterogeneous neurodevelopmental disorders not associated with clinically recognizable syndromes. By integrating short-read genome sequencing, transcriptomics, and epigenomics (DNA methylation) analyses, we resolved about 33% of the patients with heterogeneous rare neuro-developmental disorders with previous negative exome sequencing results. We also identified 13% additional candidate variants (Colin et al., 2022). This approach is effective in identifying causative variants when exhaustive clinical information is available for genotype-phenotype correlation analysis. However, additional techniques may be required to reclassify missense variants of unknown significance (VUS) or to link the presence of non-coding regulatory variants to abnormal gene expression. Blood-derived DNA methylation episignatures are highly sensitive and specific DNA methylation biomarkers allowing VUS in genes with an established episignature to be assessed or reclassified (Aref-Eshghi et al., 2020; Sadikovic et al., 2021; Levy et al., 2022), along with additional omics technologies such as proteomics analyses performed in relevant clinically accessible samples/cell lines (Alston et al., 2021), are promising complementary approaches to be used. Recently, global chromosome conformation capture techniques (Hi-C) have been also used to reclassify structural variants of unknown significance in unsolved rare-disease patients (Melo et al., 2020) allowing researchers to identify, in some cases non-coding regulatory elements triggering the ectopic expression of candidate genes (de Bruijn et al., 2020). The implementation of these approaches in diagnostics will require further investigation and validation from independent laboratories.

## Data Availability

The datasets presented in this study can be found in online repositories. The names of the repository/repositories and accession number(s) can be found below: NCBI ClinVar, accession numbers: SUB12126248, SUB12113566, SUB12114050.
