# Supplementary figures and images for: Stepwise use of genomics and transcriptomics technologies increases diagnostic yield in Mendelian disorders
Source: Front Cell Dev Biol. 2023 Feb 28;11:1021920. doi: 10.3389/fcell.2023.1021920 (PMC10011630; doi:10.3389/fcell.2023.1021920)

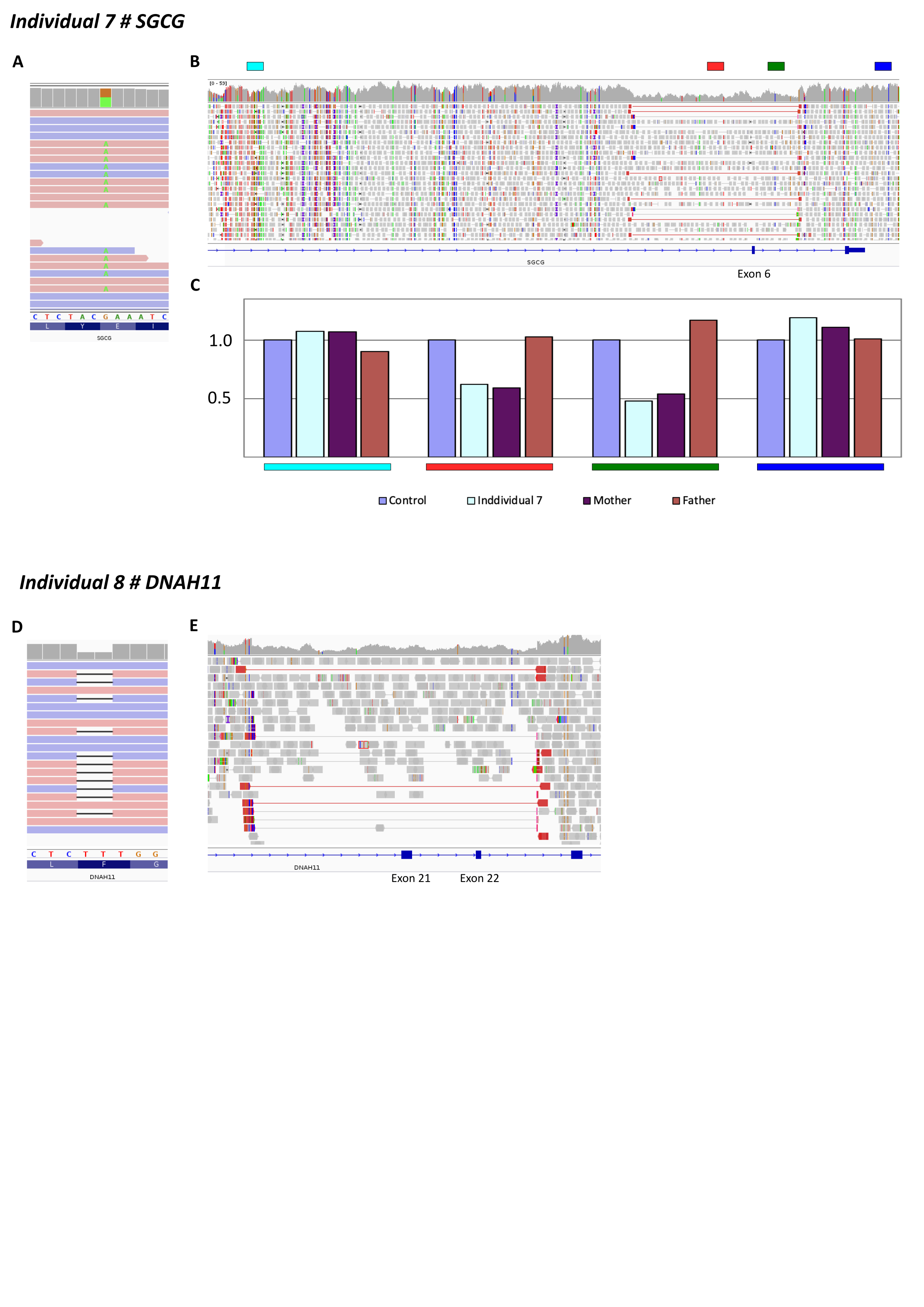

Supplement: Supplementary file 1 [file Image3.TIFF]

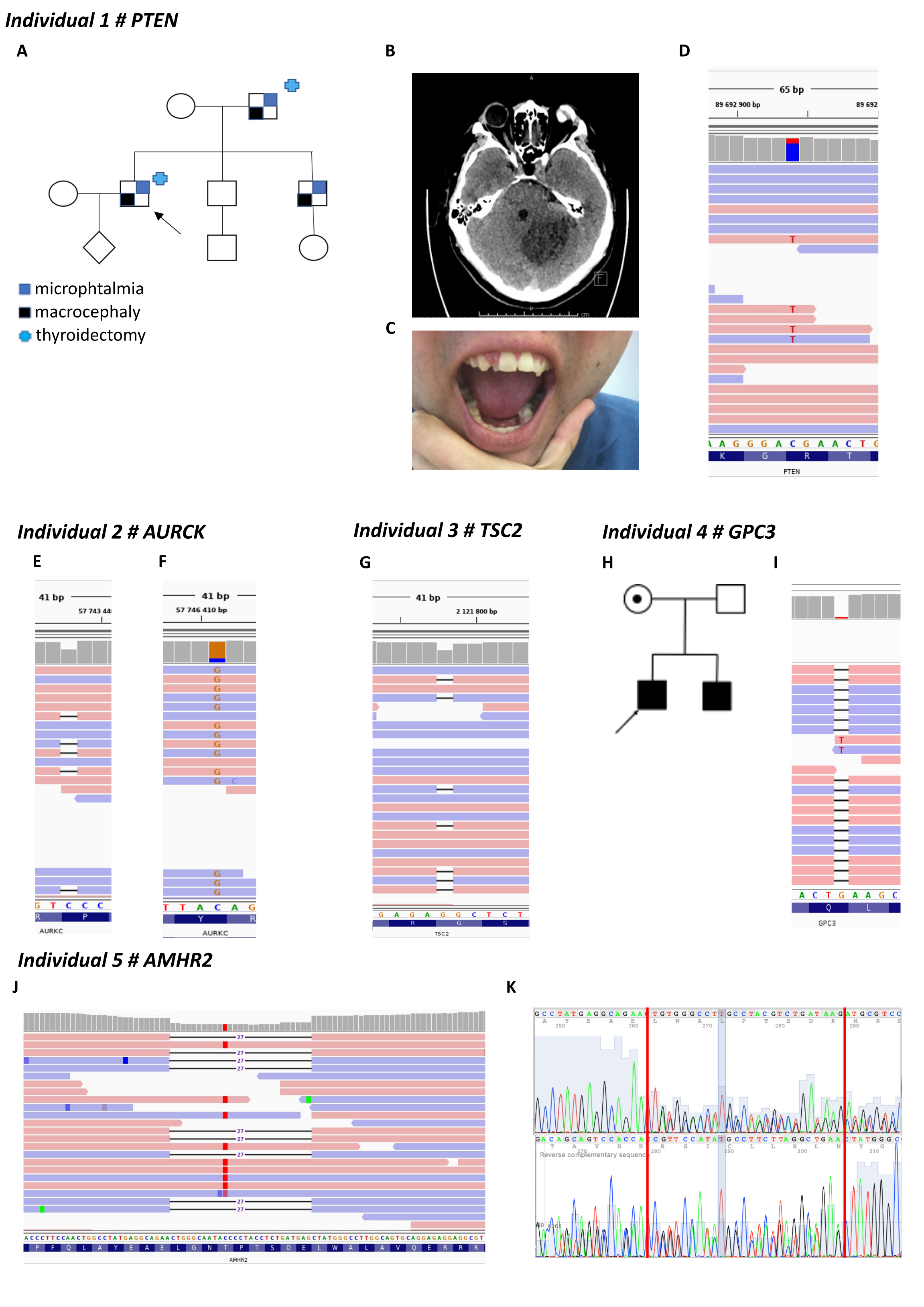

Supplement: Supplementary file 2 [file Image1.TIFF]

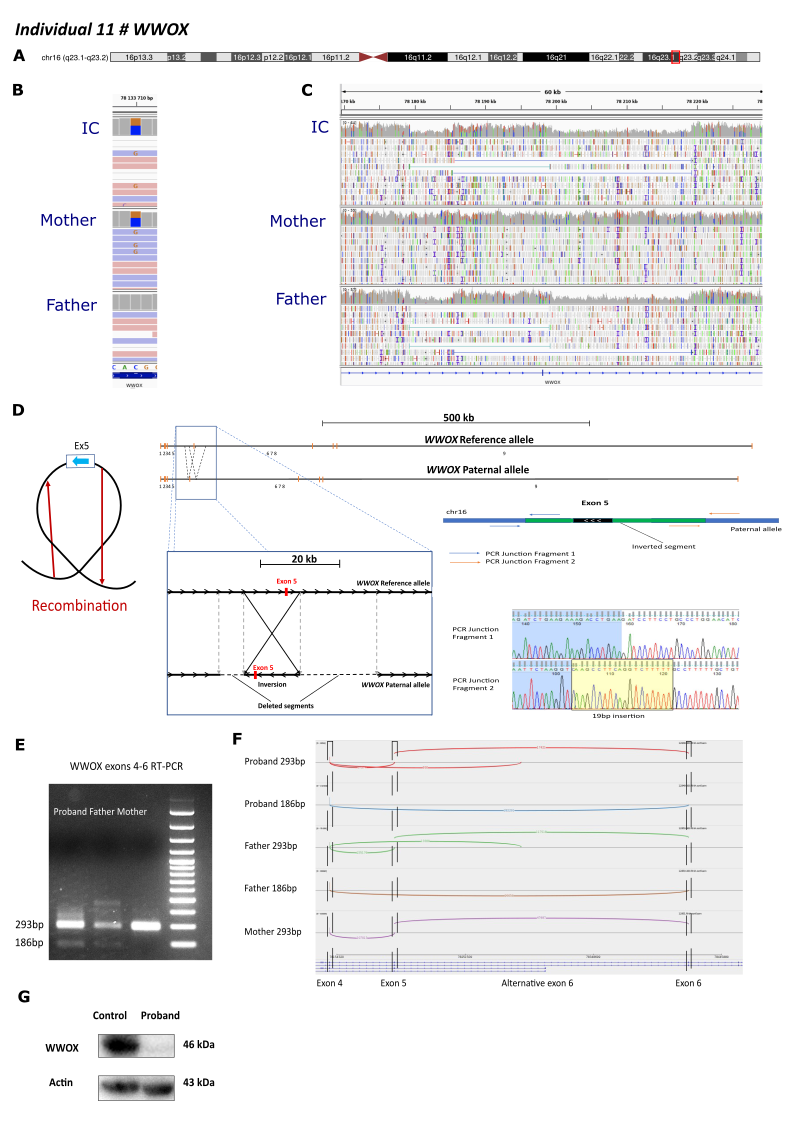

Supplement: Supplementary file 3 [file Image5.TIFF]

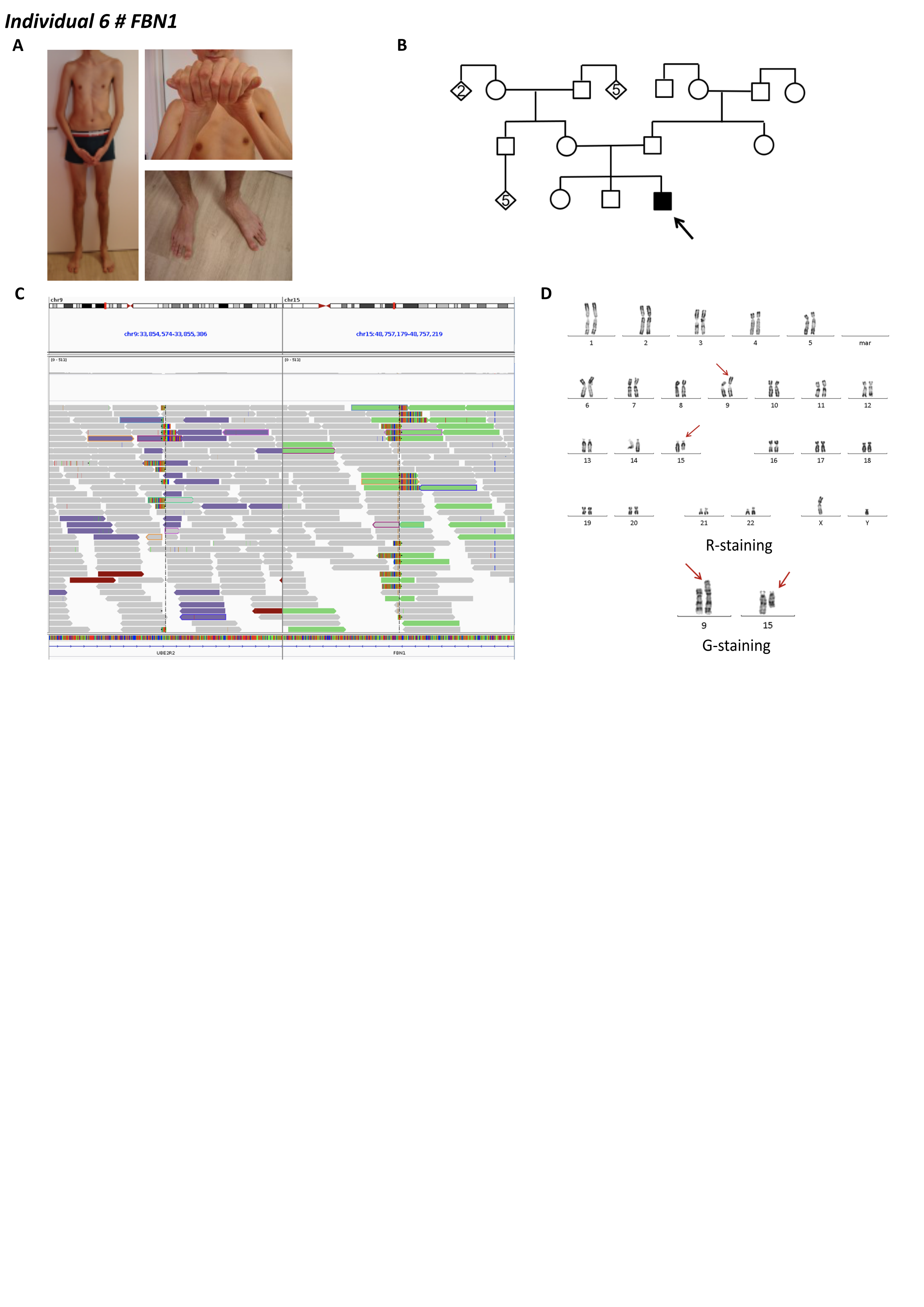

Supplement: Supplementary file 7 [file Image2.TIFF]

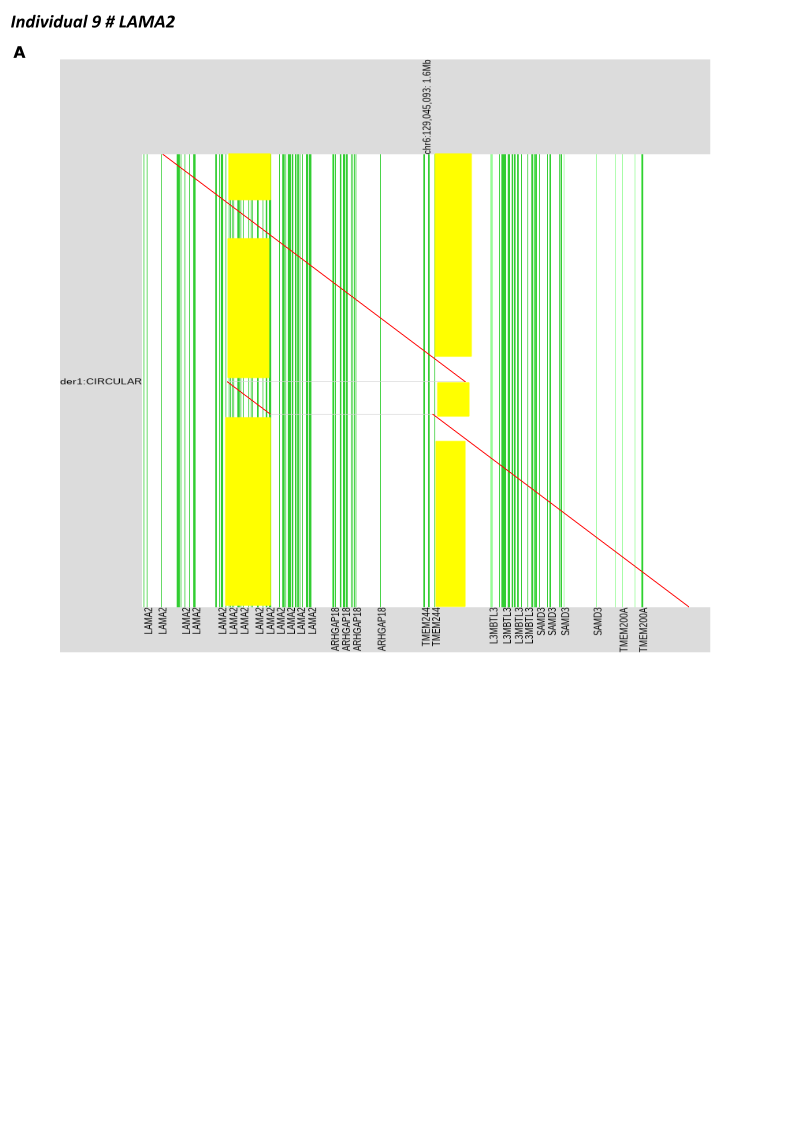

Supplement: Supplementary file 8 [file Image4.TIFF]
