# Supplementary material for: Stepwise use of genomics and transcriptomics technologies increases diagnostic yield in Mendelian disorders
Source: Front Cell Dev Biol. 2023 Feb 28;11:1021920. doi: 10.3389/fcell.2023.1021920 (PMC10011630; doi:10.3389/fcell.2023.1021920)
Supplement: Supplementary file 4 [file DataSheet1.doc]

**Legends to Figures and Tables**

**Figure S1:**

**Individual 1 -** **Cowden syndrome,MIM 158350.** **(A)** Pedigree of individual 3’s family. Macrocephaly is represented by a black square, thyroidectomy by a blue star and microphthalmia by a blue square. **(B)** Cerebral tomodensitometry of individual 1 showing a cerebellar lesion suggestive of Lhermitte-Duclos Disease. Anatomopathological examination confirmed a dysplastic cerebellar gangliocytoma. **(C)** Papillomas on hypertrophic gingival mucosa. **(D)** IGV (integrative genomics viewer) visualization of the heterozygous p.(Arg303*) variant in *PTEN*. **Individual 2 - Spermatogenic failure 5, MIM 243060**. **(E)** IGV visualization of the heterozygous p.(Leu49Trpfs*23) variant in exon 3 of *AURKC*. **(F)** IGV visualization of the heterozygous p.(Tyr248*) variant in exon 6 of *AURKC***. Individual 3 - Tuberous sclerosis complex, MIM 613254**. **(G)** IGV visualization of the heterozygous p.(Gly654Alafs*44) variant in exon 19 of *TSC2*. **Individual 4 - Simpson-Golabi-Behmel syndrome, type 1, MIM 312870. (H)** Pedigrees of individual 4’s family. The affected status is indicated by filled symbols; the carrier is indicated by a dot symbol. **(I)** IGV visualization of the hemizygous p.(Gln94Serfs*10) variant in exon 2 of *GPC3*. **Individual 5 - Persistent Mullerian duct syndrome, type II, MIM 261550.** **(J)** IGV visualization of GS results with the recurrent 27-bp deletion in exon 10 in one allele and the second heterozygous pathogenic variant in the other allele within this deletion. **(K)** Representative chromatogram showing in red lines the position of the 27-bp deletion in exon 10 and within the deletion the missense variant p.(Thr447Ile).

**Figure S2: Individual 6 - Marfan syndrome, MIM 154700.** **(A)** Photographs of individual 6. Dysmorphic features showing pectus excavatum, dolichostenomelia, arachnodactyly, thumb sign, pes cavus. **(B)** Pedigree of individual 6’s family. The affected status is indicated by a filled symbol. **(C)** IGV visualization of GS results with a balanced translocation involving chromosomes 9 and 15 with a breakpoint in the *FBN1* gene, mapping in intron 40. Soft-clipped reads aligning to another chromosome are visible. **(D)** Karyotype analysis of individual 6. Red arrows indicate the translocation between chromosome 9 and chromosome 15.

**Figure S3: Individual 7 - Muscular dystrophy, limb-girdle, autosomal recessive 5, MIM 253700.** **(A)** IGV visualization of pathogenic heterozygous p.(Glu263Lys) variant in exon 8 of *SGCG*. **(B)** IGV visualization of GS results with a heterozygous intragenic deletion of about 6.5 kb encompassing exon 6 of *SGCG*. The rectangles above the IGV image indicate the position of the primers used for independent validation. **(C)** qPCR and segregation analysis of the deletion encompassing exon 6. The tested regions follow the color scheme indicated in panel B.  **Individual 8 - Primary ciliary dyskinesia-7 (CILD7), MIM 611884.** **(D)** IGV visualization of the heterozygous p.(Phe2214Trpfs*35) variant in exon 40 of *DNAH11.* **(E)** IGV visualization of GS results with a heterozygous intragenic deletion of about 4.2 kb encompassing exons 21 and 22 of *DNAH11*.

**Figure S4: Individual 9 - Muscular dystrophy, limb-girdle, autosomal recessive 23, MIM 618138. (A)** Visualization of long-read sequencing results (Oxford Nanopore technology) with Mitsuhashi pipeline dnarange (Mitsuhashi *et al.* Genome Med. 2020 Jul 31;12(1):67). Dotplot of derivative (vertical) versus ancestral/reference (horizontal) chromosomes showing a complex rearrangement in chromosome 6.

**Figure S5: Individual 11 - Developmental and epileptic encephalopathy 28, MIM 616211. (A)** Ideogram showing chromosome 16 and *WWOX* localization. **(B)** IGV visualization of GS results with the missense variant p.(Thr12Arg) in exon 1 of *WWOX* inherited from the mother. **(C)** IGV visualization of GS results with two intronic deletions (introns 4 and 5) associated with an inversion of exon 5 in individual 11, inherited from the healthy father. **(D)** Model of the event leading to the paternally inherited complex genomic variant (left). Genomic confirmation of the structural variant identified by GS by PCR analysis (right). PCR primer design and Sanger sequencing for the characterization of the junction fragments in introns 4 and 5 of *WWOX*. **(E)** RT-PCR amplification of exons 4 to 6 showing the expected normal transcript (293 pb) and the mutated transcript (186 pb). **(F)** Sashimi plot after high throughput cDNA sequencing revealing exon 5 skipping in individual 11 and her father. **(G)** Western blot analysis showing the lack of expression of the 46 kDa band corresponding to WWOX, in the patient.
