# Supplementary material for: Stepwise use of genomics and transcriptomics technologies increases diagnostic yield in Mendelian disorders
Source: Front Cell Dev Biol. 2023 Feb 28;11:1021920. doi: 10.3389/fcell.2023.1021920 (PMC10011630; doi:10.3389/fcell.2023.1021920)
Supplement: Supplementary file 5 [file DataSheet3.doc]

**SUPPLEMENTAL MATERIAL AND METHODS**

**Cell cultures**

Fibroblasts from healthy donor and individual 13 were obtained after consent for skin biopsy. Fibroblasts were cultured in DMEM High Glucose medium (HyClone Thermo Scientific, Waltham, MA, USA) supplemented with 10% Fetal Bovine Serum (FBS, Thermo Scientific, Inc) and 1% ZellShield (Minerva, Biovalley, France). Cells were cultured at 37°C in a humidified 5% CO_2_ atmosphere.

Transplanting and seeding were performed weekly as followed: cultures were washed with PBS (5mL) before being trypsinated (4 mL) and let to incubate for 5 minutes. Trypsin was then inhibited thanks to fetal veal serum by adding culture medium (8 mL). The resulting product was then centrifugated for 5 minutes at 500G and cell pellet was resuspended. Cell count was done thanks to a Malassez device with Trypan blue.

**Total protein extraction and dosage**

Once washed with cold PBS, each well was thoroughly scraped with 100µL RIPA and lysate was recovered and let to incubate on ice for 15 minutes before centrifugation for 15 minutes at 4°C and 16 000G. The final lysate was then transferred on a clean vial and stored at -20°C.

Protein dosage was performed using a BSA calibration scale (0, 50, 100, 200, 300, 400, 500 and 700 µg/mL) from the BCA protein dosage kit (Thermo Scientific©, Waltham, USA) and a 96-well-plate spectrophotometer reader Multiskan Go™ (Thermo scientific©) by measuring samples’ absorbance at 562 nm. In brief, the BCA reagent (bicinchoninic acid) forms a colored complex with Cu_1_ ions that has a 562 nm absorbance peak. This kit contains a BCA/Cu_2_ mix that will be reduced to Cu_1_ in presence of protein in an alkaline medium. Absorbance at 562 nm is then proportionate to the amount of protein in the medium.

**Western Blot analyses**

An equal quantity of protein (patients and control) was denaturized at 95°C for 5 minutes in Laemmli buffer containing β-mercaptoethanol (355mM final, Bio-Rad©, Hercules, California, USA). Proteins were run on a 10% SDS-PAGE in 25 mM Tris, 192 mM Glycine (and 1% SDS) at 100V for migration. This latter was made with 5% or 10% acrylamide.

Transfer was then performed from migration gel to PVDF Immobilon-P membrane (Millipore©) previously activated in methanol, in a buffer containing 6g/L Trisand 3g/L boric acid at 100V during one hour at 4°C. Membranes were then saturated in a washing buffer (PBS tween 0.05%) containing 5% non-fat dried milk during 45 minutes. Membranes were blotted overnight at 4°C with specific antibodies (1/1000) in PBS 0.05% Tween, 5% milk. Membranes were then washed 3 times in PBS 0.05% Tween and probed with an anti-rabbit for WWOX (Atlas Antibodies, Inc, reference HPA023822) or anti-mouse antibody for actin (Sigma, Inc, reference 073M4760V) coupled to horseradish peroxidase. The labeled proteins were detected using the Clarity™ Western ECL substrate (Bio-Rad Laboratories, Inc) according to the manufacturer’s recommendations and visualized using ChemiDoc™ Imaging System (Bio-Rad Laboratories, Inc). Vinculin was used as cytoplasmic loading control.

**Total RNA extraction and cDNA synthesis**

RNA extraction was either performed from cell cultures or from blood stored in PAXgene Blood RNA Tubes (Qiagen - Cat. No. 762165). Cell cultures were washed in PBS and lysed in 1mL Trizol (Ambion). 200µL of chloroform was added, mixed, and incubated at room temperature (RT) for 3 minutes before centrifugation for 15 minutes at 4°C at 12 000G. The RNA-containing supernatant was then retrieved and 500µL of isopropanol were added and mixed before being incubated for 15 minutes at room temperature and then centrifugated at 4°C at 12000 G. The RNA-containing pellet was then washed twice with 75% ethanol and centrifugated (5 minutes,7500 G, 4°C). The ethanol was then carefully eliminated and the resulting dried-up pellet was resuspended in 20µL nuclease-free water.

Total RNA from blood was obtained using PAXgene Blood RNA kit (Qiagen – Cat. No. 761133) following the manufacturer’s protocol.

RNA concentration was measured out on a Multiskan Go™ (Thermo Scientific, Waltham, MA, USA) via absorbance reading à 260nm. The purity of nucleic acid was verified thanks to the evaluation of the absorbance ratio A260/A280 which evaluates protein contamination, and the A260/A230 ratio which evaluates the organic solvent contamination. The sample is considered pure if both ratios are between 1.8 and 2. cDNA was obtained using the QuantiTect Reverse Transcription kit (Qiagen GmbH, Hilden, Germany).

**PCR, qPCR and Targeted amplicon cDNA sequencing**

Primer pairs for PCR analysis, quantitative PCR (qPCR) or cDNA amplicon sequencing were designed using the Primer 3 software (https://primer3.ut.ee).

| Sample type - technique | Primer | Sequence |
| --- | --- | --- |
| Genomic DNA - qPCR | SGCG int6-7 F1 | TGCTGATGTGGGAGTGAGATT |
| Genomic DNA - qPCR | SGCG int6-7 R1 | GGCTTGCAGGTCATGATTTCT |
| Genomic DNA - qPCR | SGCG int7-8 F2 | CCCATGAAACCCTAGCCTACA |
| Genomic DNA - qPCR | SGCG int7-8 R2 | AAAGGGCAGTAGGAAGCAGAA |
| Genomic DNA - qPCR | SGCG int8 F3 | AGATTCCATCACGCTAGTCAGA |
| Genomic DNA - qPCR | SGCG int8 R3 | TCATTGTGACTTTGGCTTGTGT |
| Genomic DNA - qPCR | SGCG CTRL F4 | GTAGGCCACCCACTCACTTT |
| Genomic DNA - qPCR | SGCG CTRL R4 | AAAGTTGCAGTGCTGTTGGT |
| Genomic DNA - PCR | GPC3_ASa_F | CTCAGTGAATTTGGGCCTCC |
| Genomic DNA - PCR | SLC25A43a_R | TGGTACTCTTCCCCGCAAAT |
| Genomic DNA -PCR | SLC25A43b_F | TGGCAGAACAGGAAGAACATC |
| Genomic DNA - PCR | GPC3_Sb_R | TGGTAGTGTGGGGTTGGAAG |
| Genomic DNA - PCR | NBPF1_ex18F | GGGGCGAATTGAAAAGATGAAAG |
| Genomic DNA - PCR | NBPF1_ex18R | ACTTCCTTGATGTGCCATTGAG |
| cDNA - qPCR | GPC3_cDNA5'F | GGGTAGCAGCACGTCTCTT |
| cDNA - qPCR | GPC3_cDNA5'R | TAGGAGAGCGCGGGAGAG |
| cDNA - qPCR | GPC3_cDNA3'F | ATTCTCCTATGTTTGGCTGCTAG |
| cDNA - qPCR | GPC3_cDNA3'R | GAAATCCATGCAAAGAGAGAACG |
| cDNA - qPCR | GAPDH-7F | TGCACCACCAACTGCTTAGC |
| cDNA - qPCR | GAPDH-8R | GGCATGGACTGTGGTCATGAG |
| Genomic DNA - PCR | WWOX_INV_ex5_1F | AAAATAGAGAAGCCGATAGTGTAGC |
| Genomic DNA - PCR | WWOX_INV_ex5_1R | CCACTGTCTTTGTAGAGCTTTCAG |
| Genomic DNA - PCR | WWOX_INV_ex5_2F | GGTATTTCCTGAGCATCCTGATTTA |
| Genomic DNA - PCR | WWOX_INV_ex5_2R | TCCTTCCTATCAGATCATCTTCGAA |
| cDNA - PCR | WWOX_cDNA_ex4-6F | AAGACTGGCGTTTACTGTGGAT |
| cDNA - PCR | WWOX_cDNA_ex4-6R | GAGCGAGGTCCAGGGTCATT |

Quantitative PCR analysis was performed according to the manufacturer's recommendations with QuantiTect SYBR Green PCR kit (Qiagen, Courtaboeuf, France) on a LightCycler® 480 Instrument II, using 96-well plates (Roche Applied Science, Indianapolis, Indiana, USA).

PCRs were performed with the Prime Star GXL DNA polymerase kit (Takara Bio Inc​) using the following touchdown conditions: initial denaturation 98°C for 3 min; 5 thermal cycles of denaturation at 94°C for 30 sec, primer annealing 65°C for 30 sec, primer extension 68°C for 1 min per Kb; 35 thermal cycles using 60°C instead of 65°C for primer annealing; followed by a final incubation at 10 °C.

PCR products were verified by agarose gel electrophoresis using the ladder GeneRuler 1kb Plus (Thermo Scientific- Cat. N° 11581625), and amplicons suitable for next-generation sequencing analysis were purified with AMpure XP magnetic beads ([Beckman Coulter](https://www.beckman.fr/) Inc., Brea, CA, USA). Sequencing libraries were prepared with the Nextera XT kit (Illumina, San Diego, CA, USA) and sequenced on the Illumina Miseq using a paired-end protocol (2x150 bases in length).

RNA sequencing data were aligned on the human genome reference (GrCh37/Hg19) using the STAR aligner (version 2.5.2)^53^ for each sample.

Data analysis was performed using direct inspection of spliced reads in the Integrative Genomics Viewer software (Broad Institute) and Sashimi plots were generated in Python (2.7.18) using the package ggsashimi (0.6.0).
