# Supplementary material for: Stepwise use of genomics and transcriptomics technologies increases diagnostic yield in Mendelian disorders
Source: Front Cell Dev Biol. 2023 Feb 28;11:1021920. doi: 10.3389/fcell.2023.1021920 (PMC10011630; doi:10.3389/fcell.2023.1021920)
Supplement: Supplementary file 6 [file DataSheet2.docx]

**Supplementary data**

***Individual 1 # PTEN***

Individual 1 was a 30-year-old male, the third child of non-consanguineous French parents. He was referred to a geneticist during the first pregnancy of his wife due to left microphthalmia with coloboma. He also presented with macrocephaly, oral papillomas and dental abnormalities. He had undergone thyroidectomy following thyroid nodules. He also presented Lhermitte-Duclos disease. Immunofluorescence study of PTEN in thyroid tissue showed a clear decrease of PTEN expression. His father and one of his brothers also have macrocephaly. No familial history of neoplasia. A diagnosis of Cowden syndrome was suggested. Genetic investigations consisting of array-CGH, syndromic microphthalmia panel and ES with good coverage and depth on *PTEN* were negative.

***Individual 2 # AURKC***

Individual 2 was a 34-year-old male, from a family of seven children (including 6 boys), with no noteworthy family medical history. He presented teratozoospermia with marcrozoospermia confirmed on several sperm samples. Genetic investigations consisting of *AURKC* Sanger sequencing identified a frameshift variant p.(Leu49TrpfsTer23) in exon 3 of *AURKC*. The variant was not found in gnomAD genomes. ClinVar classifies this variant as Pathogenic and *in* *silico* prediction scores are in favor of its pathogenicity.

***Individual 3 # TSC2***

Individual 3 was a 2-year-old girl, the first child of unaffected, non-consanguineous African parents. An ultrasound during the second trimester of pregnancy detected multiple cardiac tumors suggestive of cardiac rhabdomyoma. MRI performed at 32 WG + 6 showed multiple cortical tubers and sub-ependymal nodules. Tuberous sclerosis complex (TSC) was clinically diagnosed. The child was born at 40 WG; birth length = 48 cm (16th percentile), weight = 3010 g (19th percentile) and OFC = 38 cm (99th percentile). Physical examination revealed hypomelanotic macules and lumbar shagreen patch. Asymptomatic cardiac rhabdomyoma were confirmed on the neonatal cardiac ultrasound. No renal tumor was observed on the abdominal ultrasound. Electroencephalogram at the age of 1 week showed asymmetrical tracing with spikes. Preventive antiepileptic treatment with vigabatrin was started at the age of 3 weeks. At 18 months, she walked with help. Prenatal and postnatal genetic investigations consisting of array-CGH and screening for TSC on amniotic fluid then on blood sample and saliva sample by specific panel were negative.

***Individual 4 # GPC3***

Individual 4 was a 6-year-old boy, the first child of unaffected, non-consanguineous French parents. An ultrasound during pregnancy detected omphalocele. Prenatal array-CGH was normal. The child had a premature birth at 36 WG; macrosomic birth length = 51 cm (96th percentile), weight = 3600 g (97th percentile) and OFC = 34 cm (70th percentile). A physical examination revealed macroglossia and omphalocele. He underwent visceral surgery at birth. At the age of five, dysmorphic features were observed such as epicanthus, everted lips. Physical examination revealed a supernumerary nipple. His brother presented with coarse facies, macroglossia and supernumerary nipples. Genetic investigations consisting of screening for Beckwith-Wiedemann syndrome by methylation analysis and *CDKN1C* Sanger sequencing and Simpson-Golabi-Behmel syndrome by Sanger sequencing were negative.

**Individual 5 #** ***AMHR2***

Individual 5 was a 37-year-old male, the third child of unaffected, non-consanguineous French parents, presenting with bilateral cryptorchidism for which a karyotype (normal, 46,XY) was carried out. He underwent surgery at the age of 9 months to remove one testicle; during the surgy, a uterus and fallopian tubes were observed leading to a diagnosis of Persistent Mullerian duct syndrome (PMDS). A targeted mutational screening of the *AMHR2* gene identified the most common mutation, a 27-bp deletion in the kinase domain inherited from the mother. The 5’ *AMHR2* promotor sequencing and *AMHR2* southern blot to find the second hit was negative. Sanger sequencing of *AMH* was negative.

**Individual 6 #** ***FNB1***

Individual 6 was a 23-year-old male, the third child of unaffected, non-consanguineous French parents. The pregnancy was uncomplicated with normal screening ultrasounds. He was born at 41 WG with intrauterine growth retardation, birth length (47.5 cm, 1.7th percentile), weight (2800 g, 2.4th percentile) and OFC (33 cm, 4.4th percentile). At the age of five years, he was diagnoses with Marfan syndrome due to ectopia lentis, skeletal signs (e.g. highly arched palate, retrognathia, pectus excavatum, dolichostenomelia, arachnodactyly, thumb and wrist signs, elbow flexion contracture, scoliosis, pes cavus, protrusio acetabuli), and cardiovascular signs (e.g. aortic dilatation and mitral valve prolapse). Evidence of skin stretch marks and dural ectasia appeared subsequently. Previous genetic investigations, consisting of Sanger sequencing and MLPA techniques of *FBN1*, panel genes of Marfan syndrome and related diseases, array-CGH, and ES solo were negative.

**Individual 7 #** ***SGCG***

Individual 7 was a 33-year-old male, the third child of unaffected, non-consanguineous French parents. He was a sporadic case with no family history. The pregnancy had been uncomplicated. He was born at 37 WG with normal birth length (48 cm, 45th percentile), weight (3060 g, 62nd percentile) and OFC (33.5 cm, 42nd percentile). He walked at 13 months but he never managed to run or jump. At the age of seven, his symptoms increased with frequent falls, decreased muscle strength and gait disorders. Physical examination showed waddling walk, calf muscle pseudohypertrophy, proximal muscles weakness, hyperlordosis and genu valgum. He lost the ability to walk independently at the age of 10, and cardiomyopathy (with slight dilation of the left ventricle) was diagnosed at age 21. A blood test revealed high CK level (14722 U/l). At the age of 8 years, muscle biopsy revealed a dystrophic appearance. The muscle dystrophin study was normal. Indirect immunofluorescence study of adhalin showed a marked decrease of adhalin expression in the membrane of almost all the striated muscle fibers. A western blot of dystrophin and other muscle proteins involved in progressive muscular dystrophies was carried out but the results were inconclusive. An electromyogram confirmed symptoms indicative of myopathic involvement, without involvement of the anterior horn. Previous genetic investigations, consisting of screening for pathogenic variants in alpha-sarcoglycane gene and rearrangement of the dystrophin gene were normal. WES simplex identified a pathogenic heterozygous missense variant p.Glu263Lys in *SGCG* gene previously described in literature (PMIDs 25802879, 24534832). This variant has been reported as being pathogenic in ClinVar and in HGMD databases. It is missing from the gnomAD homozygous population database. The *in*-**silico** prediction scores support the pathogenicity of this variation. Pathogenic biallelic variants of *SGCG* are responsible for autosomal recessive transmission type 2C girdle muscular dystrophy (OMIM # 253700), which belongs to the sarcoglycanopathies group. The clinical presentation of individual 7 is compatible with the clinical phenotype associated with this gene.

***Individual 8 # DNAH11***

Individual 8 was the first child of unaffected, non-consanguineous French parents. The pregnancy was marked by gestational diabetes, requiring only health and dietary guidance. At 23 WG + 2, an ultrasound revealed complex cardiac malformation consisting of a single ventricle with single atrium and unique atrioventricular valve. After counseling, the couple opted for termination of pregnancy at 24 WG + 5. A fetal autopsy revealed cardiac dextroposition, thoracic situs inversus, ambiguous abdominal situs, polysplenia and pancreatic hypoplasia. The combination of these situs abnormalities suggested Ivemark syndrome type 1.

**Individual 9 #** ***LAMA2***

Individual 9 was a 14-month-old boy, the first child of unaffected, non-consanguineous French parents. The pregnancy had been marked by 5th percentile intrauterine growth retardation. Active fetal movements were present, and the amount of amniotic fluid was normal. The child was born at 41 WG by emergency cesarean section for altered fetal heart rate and double coiling of the umbilical cord around fetal neck. Apgar's score was 7-9-10. Intrauterine growth retardation was confirmed with birth length (51 cm, 48th percentile), weight (2825 g, 2nd percentile) and OFC (33 cm, 4th percentile). He had neonatal hypotonia, joint contractures, retrognathia, poor feeding with swallowing disorder, plus hypoglycemia. Neonatal brain MRI was normal. Blood test revealed high CK level (89000 U/l at day 2 to 3000 U/l after day 5). Exhaustive metabolic tests were normal. Previous genetic investigations, consisting of Array-CGH showed deletion of exons 3 and 4 of *LAMA2* gene inherited from the father. A muscle biopsy was suggestive of congenital muscular dystrophy with negative merosine expression, in favor of a primary merosin deficit by primary impairment of merosine by pathogenic variants of the *LAMA2* gene. *LAMA2* biallelic loss of function is associated with congenital muscular dystrophy (MIM # 607855) of autosomal recessive inheritance. The described phenotypes associated with *LAMA2* range from mild congenital muscular dystrophy to severe early muscular dystrophy. The latter usually includes severe hypotonia, muscle weakness that appears in the first six months of life with eating and breathing difficulties, muscle retractions. The phenotype of individual 9 could be compatible with this syndrome.

**Individual 10 #** ***GPC3***

Individual 10 was a 5-year-old boy, the fourth child of unaffected, non-consanguineous Tunisian parents. The pregnancy of the second child had been marked by polyhydramnios, macrosomia, omphalocele, pyelic dilatation and visceromegaly. The birth was premature at 29 WG and the newborn boy died at 13 days of life. Genetic investigation, consisting of screening for Becwith-Wiedemann syndrome, was normal. The fourth pregnancy was marked by polyhydramnios, macrosomia, pyelic dilatation, visceromegaly and bowel dilatation. The child was born at 27 WG + 5 following a premature rupture of membranes. He was macrosomic with birth length at 42 cm (39th percentile), weight at 1700 g (99th percentile) and OFC at 28.5 cm (99th percentile). In the neonatal period, visceromegaly was confirmed and global hypotonia observed. Dysmorphic features were observed such as telecanthus, palpebral edema, low-set ears, wide mouth, gingival overgrowth, macroglossia, thin upper lip vermilion, smooth philtrum. Physical examination revealed supernumerary nipple, umbilical hernia, dorsal hirsutism, single left transverse palmar crease. An echocardiography was normal. An examination of the mother revealed a supernumerary nipple. The clinical diagnosis was Simpson Golabi Behmel syndrome. Targeted inspection of array-CGH results indicated a single deviating probe suggesting an intronic deletion in intron 2 of *GPC3*.

**Individual 11 #** ***WWOX***

Individual 11 was a 7-year-old girl, the second child of unaffected, non-consanguineous French parents. The pregnancy was characterized by a decrease in active fetal movements and micro-oscillating rhythm a few days before childbirth. She was born at 39 WG with a normal birth length (51 cm, 85th percentile), weight (3320 g, 61st percentile) and OFC (34.5 cm, 61st percentile). Apgar's score was 9 – 10. The onset of seizures at the age of three months was associated with severe global developmental delay and progressed to epileptic encephalopathy. Seizures were of varying types and included focal or generalized tonic-clonic seizures, myoclonic seizures, absence, atonic seizures, and automatism. She also has axial hypotonia, limb spasticity, ataxia, dysmetria, movement disorders, and stereotypies, along with language impairment. Physical examination revealed no obvious dysmorphic features, except for strabismus. Ophthalmological examination reported nystagmus. EEG records showed altered background activity with generalized slowness, and multifocal abnormalities. Absence of physiological features during the sleep with generalized discharges of spikes and hypsarrhythmia. Brain magnetic resonance showed progressive cerebral atrophy. Exhaustive metabolic tests were normal. No abnormalities in cerebrospinal fluid neurotransmitters or glycorrhachia were observed. Previous genetic investigations, consisting of Array-CGH, screening for Angelman syndrome (methylation of the *UBE3A*), mitochondrial DNA sequencing were negative. ES trio showed a missense variant p.(Thr12Arg) in *WWOX* which is absent from the GnomAD database with *in silico* prediction scores in favor of pathogenicity.

**Individual 12 #** ***VPS13B***

Individual 12 was a 30-year-old male, the child of unaffected, non-consanguineous French parents, with hypotonia and global development delay. He walked at 24 months and had delayed speech and language development. Retinitis pigmentosa was diagnosed at the age of 10. He had facial dysmorphisms including downslanting palpebral fissures, midface retrusion, short philtrum, open mouth appearance, high palate, micrognathia. Physical examination revealed truncal obesity, narrow hand with tapered fingers, and joint hyperextensibility; no microcephaly nor neutropenia. Brain MRI was normal. He was diagnosed with Cohen syndrome. A targeted mutational screening of the *VPS13B* gene identified a pathogenic heterozygous frameshift variant p.(Tyr2711*), inherited from the mother.

**Individual 13 #** ***LSS***

Individual 13 was a 4-year-old boy, the second child of non-consanguineous healthy parents, with no noteworthy familial history. During the pregnancy, a small penis was observed. Growth parameters at birth after 37 weeks of gestation were low. His weight was 2750 g (6th percentile), height 46 cm (1.7th percentile) and OFC 34 cm (30th percentile). Micropenis and alopecia were diagnosed at birth. Minor erythroderma and thin desquamation on abdomen were observed. A clinical examination at the age of 2 months showed alopecia universalis (absence of hair, eyebrows, and eyelashes). Skin and nails were normal. Micropenis was confirmed. A neurological evaluation was normal with normal tone and movements, good head control, normal eye contact. He was admitted at the age of 3 months for general non febrile repeated seizures. The interictal electroencephalogram was normal. Cerebral MRI was considered normal (slight ventricular asymmetry, Rathke’s cleft cyst). Neurological evaluation at 3 months showed severe hypotonia and absent eye contact. Seizures were controlled in four months after treatment with Levetiracetam and Valproate. At 11 months, an examination revealed poor eye contact and global psychomotor delay. He received physiotherapy and special education for children with visual impairment and psychomotricity education, and at the age of 15 months had progressed well in terms of visual abilities, motor and verbal skills. He spoke his first words at 18 months and was able to walk independently at 22 months. At the age of 3 years and 8 months, growth parameters were in the normal range. Alopecia was still complete, skills were globally delayed, with impaired coordination of movements, and wide-base gait. He could understand simple orders and speak in short sentences of two to three words. Eye contact was not perfect. He was agitated and suffered from attention deficit. He began school and received help for special needs. Some features of autism spectrum disorder were present. Sanger sequencing identified only one variant c.1955C>T; p.(Thr652Ile) in exon 20 of *LSS*. This maternally inherited missense variant is absent from the gnomAD database. Linkage analysis using single nucleotide polymorphisms of *LSS* revealed that affected individual 13 and unaffected sister had inherited the same paternal allele but had inherited a different allele from their mother. However, the authors observed that the transcript corresponding to the paternal allele was less expressed than the one related to the alternative allele. RT-PCR from fibroblast RNA revealed an allelic imbalance noticeable in individual 13 and his father. The cause of this allelic imbalance was unexplained.

**Individual 14 # *APC***

Individual 14 was a 34-year-old female, the third child of unaffected, non-consanguineous French parents. A colonoscopy performed at the age of 28 due to abdominal pain revealed multiple colonic and rectal polyposis and adenocarcinoma of the transverse colon. The presence of polyposis at this young age suggested a need for genetic testing for a predisposition to polyposis. A targeted mutational screening of *APC* and *MYH* was performed on blood samples and digestive tract biopsies; gene panels of familial adenomatous polyposis and ES were negative. RER phenotyping of the tumor showed revealed an MSS phenotype and, in terms of immunohistochemistry, persistent expression of hMLH1, hMSH2, hMSH6 and PMS2 proteins was observed.

***Individual 15 # ANKRD11***

Individual 15 was a 22-year-old male, the first child of a single French mother. The pregnancy was marked by intrauterine growth retardation, oligohydramnios and cytomegalovirus seroconversion. Prenatal karyotype was normal. The birth was premature at 31 WG. Intrauterine growth retardation was confirmed with birth length at 31 cm (<1 percentile), weight at 650 g (<1 percentile) and OFC at 24,8 cm (<1 percentile). Inguinal hernia surgery was performed during the neonatal period. His feeding was poor, and he still needed a gastrotomy. All milestones of motor development were delayed. He walked at 2 years of age. He has speech and language impairment. He has attention deficit hyperactivity disorder, behavioral abnormality such as autistic behavior and aggressive behavior. He has a sleep disturbance. Myoclonic seizures were diagnosed at the age of 4. He has facial dysmorphisms including triangular face, large forehead with low anterior hairline, broad eyebrow, large prominent ears, long eyelashes, synophrys, telecanthus, upslanted palpebral fissure, prominent nose, anteverted nares, wide mouth, gingival overgrowth, high palate, retrognathia, incisor macrodontia. Physical examination revealed microcephaly (<-3SD), short stature (<-2SD), decreased body weight (<-2SD), thick hair, Cafe-au-lait spot on the back, flat thumbs, clinodactyly and camptodactyly of the 5th finger, drooling and tip-toe gait. Paraclinical examinations showed left pelvic kidney and right renal malrotation, delayed skeletal maturation, C2-C3 vertebral fusion, six lumbar vertebrae, hearing impairment, and cerebral atrophy on brain MRI. A diagnosis of KBG syndrome was suggested. Previous genetic investigations, consisting of array-CGH, intellectual disability panel with *ANKRD11* gene, and solo ES yielded normal results.
